# GMP-compliant iPS cell lines show widespread plasticity in a new set of differentiation workflows for cell replacement and cancer immunotherapy

**DOI:** 10.1093/stcltm/szae047

**Published:** 2024-07-23

**Authors:** Daniel Terheyden-Keighley, Melanie Hühne, Thomas Berger, Björn Hiller, Soraia Martins, Anna Gamerschlag, Davood Sabour, Andrea Meffert, Andreas Kislat, Carsten Slotta, Francois Hafezi, Jens Lichte, Smita Sudheer, Karen Tessmer, Katherina Psathaki, Marius Ader, Gesine Kogler, Boris Greber

**Affiliations:** Catalent Düsseldorf GmbH, 40764 Langenfeld, Germany; Catalent Düsseldorf GmbH, 40764 Langenfeld, Germany; Catalent Düsseldorf GmbH, 40764 Langenfeld, Germany; Catalent Düsseldorf GmbH, 40764 Langenfeld, Germany; Catalent Düsseldorf GmbH, 40764 Langenfeld, Germany; Catalent Düsseldorf GmbH, 40764 Langenfeld, Germany; Catalent Düsseldorf GmbH, 40764 Langenfeld, Germany; Catalent Düsseldorf GmbH, 40764 Langenfeld, Germany; Catalent Düsseldorf GmbH, 40764 Langenfeld, Germany; Catalent Düsseldorf GmbH, 40764 Langenfeld, Germany; Catalent Düsseldorf GmbH, 40764 Langenfeld, Germany; Catalent Düsseldorf GmbH, 40764 Langenfeld, Germany; Catalent Düsseldorf GmbH, 40764 Langenfeld, Germany; Center for Regenerative Therapies Dresden (CRTD) and Center for Molecular and Cellular Bioengineering, Dresden University of Technology, 01307 Dresden, Germany; Center for Cellular Nanoanalytics (CellNanOs), University of Osnabrück, 49076 Osnabrück, Germany; Center for Regenerative Therapies Dresden (CRTD) and Center for Molecular and Cellular Bioengineering, Dresden University of Technology, 01307 Dresden, Germany; Institute of Transplantation Diagnostics and Cell Therapeutics and Jose Carreras Stem Cell Bank, University Hospital of Düsseldorf, 40225 Düsseldorf, Germany; Catalent Düsseldorf GmbH, 40764 Langenfeld, Germany

## Abstract

Cell therapeutic applications based on induced pluripotent stem cells (iPSCs) appear highly promising and challenging at the same time. Good manufacturing practice (GMP) regulations impose necessary yet demanding requirements for quality and consistency when manufacturing iPSCs and their differentiated progeny. Given the scarcity of accessible GMP iPSC lines, we have established a corresponding production workflow to generate the first set of compliant cell banks. Hence, these lines met a comprehensive set of release specifications and, for instance, displayed a low overall mutation load reflecting their neonatal origin, cord blood. Based on these iPSC lines, we have furthermore developed a set of GMP-compatible workflows enabling improved gene targeting at strongly enhanced efficiencies and directed differentiation into critical cell types: A new protocol for the generation of retinal pigment epithelium (RPE) features a high degree of simplicity and efficiency. Mesenchymal stromal cells (MSCs) derived from iPSCs displayed outstanding expansion capacity. A fully optimized cardiomyocyte differentiation protocol was characterized by a particularly high batch-to-batch consistency at purities above 95%. Finally, we introduce a universal immune cell induction platform that converts iPSCs into multipotent precursor cells. These hematopoietic precursors could selectively be stimulated to become macrophages, T cells, or natural killer (NK) cells. A switch in culture conditions upon NK-cell differentiation induced a several thousand-fold expansion, which opens up perspectives for upscaling this key cell type in a feeder cell-independent approach. Taken together, these cell lines and improved manipulation platforms will have broad utility in cell therapy as well as in basic research.

## Graphical Abstract

**Graphical Abstract:**
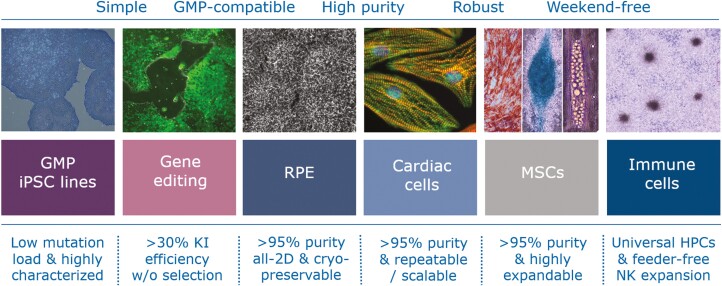

**Significance Statement**

Human pluripotent stem cells are highly versatile in that they can be converted into various desired cell types of the body and this potential may be exploited for novel medical treatments. For example, heart muscle cells derived from these stem cells may be used for replacing damaged cells upon myocardial infarction whereas immune cells may be programmed to destroy cancer cells in a patient’s body. This work introduces a new set of pluripotent stem cell lines that can be used for clinical applications and establishes a new set of tools to convert these cells into key cell types for therapy.

## Introduction

Cell therapeutic approaches may roughly be classified by their mode of action: Transplanted cells like mesenchymal stromal cells (MSCs) may exert beneficial effects such as immunomodulation in a rather undefined and paracrine manner. Alternatively, therapeutic cells can serve to specifically replace degenerated or otherwise damaged tissue and its function. Thirdly, cells may be engineered to actively fight disease by releasing specific factors or kill unwanted cells as in cancer immunotherapy. iPSCs as an emerging modality and universal starting point may serve to generate cells for all these needs.^1,2^

Fundamentally, that promise resides in the defining properties of iPS cells, self-renewal capability—as needed for clonal isolation and expansion—and pluripotency as a basis for generating virtually any cell type at will. Indeed, reliable culture conditions and dedicated differentiation protocols based on iPSCs have been established over the years and have been entering the first clinical trials.^3^ Viable examples include workflows for creating retinal pigment epithelium (RPE), the derivation of MSCs, cardiomyocyte differentiation, or the scalable generation of engineered NK cells.^4-8^ Moreover, CRISPR/Cas-based gene editing technology as well as the idea of generating HLA-homozygous iPS cells from corresponding donors have opened perspectives for allogeneic cell therapy with a reduced need for immune suppression in patients.^9,10^

Current guidelines for good manufacturing practice (GMP) impose strict regulations and, thereby, cost pressure on the manufacture of advanced therapeutic products (ATMPs). These are essential to ensure patient safety and ultimately efficacy. Many contemporary iPSC manipulation protocols originating in academic settings are not able to cope with these requirements including the expectations to qualify and test every supplier, device, and critical reagents. Another major concern is interexperimental consistency as failed GMP batches trigger lengthy investigations and corrective actions. Thus, the selection for simplicity in ATMP manufacturing workflows is instrumental.

Overall, one or more of the following applies to most available differentiation protocols: complicated handling steps or number of reagents used; technically inefficient or inconsistent due to partial reliance on spontaneous differentiation or on selective—rather than instructive—mechanisms; usage of undefined, complex, or even animal-derived components causing risk; implying hard-to-qualify or GMP-incompatible cell enrichment and selection steps; or increased cost of goods and process duration.

To address this need, we have generated iPS cell lines resulting from the formal validation of a GMP-compliant manufacturing process. Furthermore, we have developed a new set of differentiation protocols as well as an improved gene editing procedure that overcome several of the above shortcomings in each case. Thus, we demonstrate the widespread compatibility of these manipulation platforms with independent GMP iPS cell lines.

## Materials and methods

A detailed description of methods can be found in Supplementary Information.

### Study design

iPSCs were manufactured in a GMP-certified clean room facility. A manufacturing authorization was granted by the regulatory authorities (Bezirksregierung Düsseldorf and Paul Ehrlich Institute) with regard to the validated iPSC process and analytics. Batch records and process documentation templates were designed to also meet several FDA-specific GMP requirements. QC specifications are summarized in Supplementary Table S1.

### Reprogramming process and QC

iPSCs were generated from selected cord blood units based on informed re-consenting of the donors. The preceding process of CD34^+^ isolation and amplification has been described elsewhere.^11^ Episomal reprogramming was technically related to Okita et al^12^ while replacing dominant negative *TP53* with a proprietary factor to avoid safety concerns. Selected candidate clones were cryopreserved as prospective GMP banks following vector clearance. Validated analytical testing is summarized in Supplementary Table S1 and was based on standard methods in case of in-house assays,^13,14^ or based on outsourcing to qualified suppliers.

### iPSC differentiation

Reagents used for iPSC manipulation were selected to also be available as GMP products or allow being qualified accordingly. Prior to differentiation, iPS cells were maintained in StemMACSiPS-Brew XF medium (Miltenyi Biotec) on iMatrix-511 (Amsbio). iPSCs under R&D conditions were routinely replated twice per week in the presence of 10 µM Y-27632^15^ using Accutase (Merck). By convention, the first day of a given treatment was termed day 0.

### RPE differentiation

Basic RPE medium consisted of 15% (v/v) Knockout serum replacement in DMEM/F12 with 1× Glutamax (Thermo Fisher). The optimized induction cocktail was composed of 1 µM PD0325901, 5-10 µM SB431542, 0.25 µM Dorsomorphin, and 25 ng/mL Activin A, followed by ActA alone.

### MSC differentiation

CHIR99021 (4 µM) in iPS-Brew medium was administered to low-density iPSC cultures for 6 days, followed by switching to MSC media (“RB” = RoosterNourish-MSC-XF, RoosterBio; “MB” = StemMACS MSC-XF, Miltenyi Biotec).

### Cardiac differentiation

Starting from dissociated iPSCs in 6-well format, the differentiation medium was Knockout DMEM (Thermo Fisher) with 0.1% (w/v) HSA, 250 µM 2-phospho-ascorbate, and L-glutamine, plus stage-specific factors/additives including 3-5 ng/mL BMP4, 1 µM CHIR99021, 7.5 ng/mL FGF2, and 7.5 ng/mL Activin A on day 0; 0.5 µM C-59 from days 2-3.

### Hematopoietic induction

**Figure 5.**
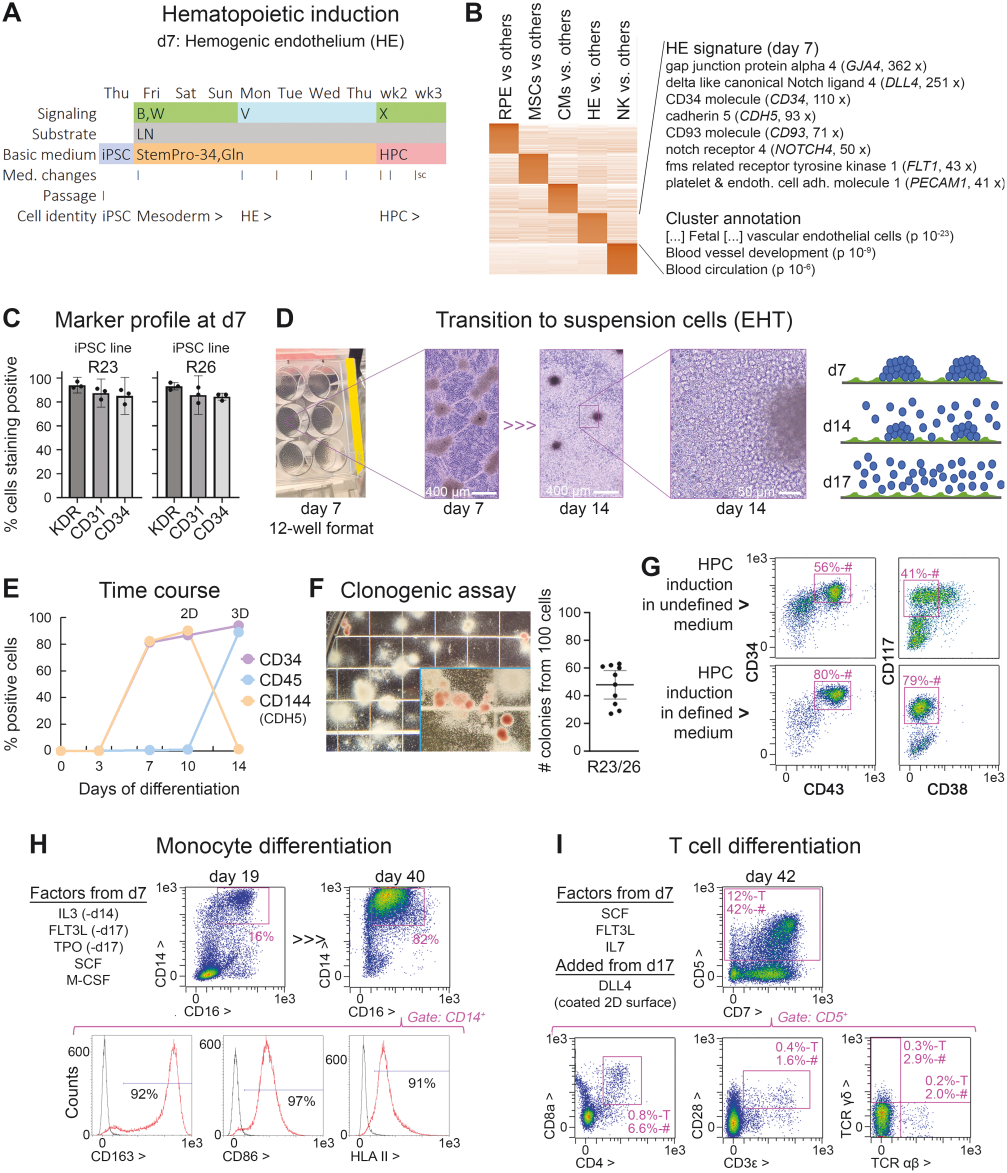
Universal immune cell differentiation platform based on the induction of an HPC intermediate state. (A) 2D protocol for the induction of HPCs by days 14-17. (B) W = BMP and WNT signaling stimulation; V = VEGFA; S, X = target cell-dependent factors such as SCF (see Methods). After 1 week, the cells resembled hemogenic endothelium-like precursors (HE). (B) Expression profiling of the day 7 cells indicates a hybrid signature of endothelial and hematopoietic precursor cells. (C) Flow cytometry data of day 7 cells suggesting near-homogeneous differentiation into HE-like cells (*n* = 3). (D) Cellular morphology in experimental 12-well format and illustration of EHT process. (E) Representative time course data (flow cytometry) highlighting a homogeneous transition into hematopoietic suspension cells after day 7 (CD34^+^/CD45^+^), at the expense of endothelial commitment marked by VE-cadherin. (F) Methylcellulose assays using independent iPSC lines indicate robust formation of hematopoietic colonies from days 14 to 17 cells, comparable to primary HSCs. (G) Expression signature of iPSC-derived HPCs depends on basic medium used, with defined conditions giving rise to naïve CD34^+^/CD43^+^/CD117^+^/CD38^−^ cells. (H) Proof-of-concept experiment demonstrating directed differentiation competence of day 14 cells into monocytes (myeloid lineage). (I) Proof-of-concept experiment demonstrating directed differentiation competence of iPSC-derived HPCs into immature T cells marked by CD4 and 8a. A small fraction also showed mature alpha beta T-cell receptor expression (bottom right panel).

iPSCs were reseeded at 200 k per 12-well and treated with 25 ng/mL BMP4 and 8 µM CHIR99021 in StemPro-34 medium (Thermo Fisher) for 72 hour and, from days 3-6, with 200 ng/mL VEGFA and 10 µM SB431542 (Figure 5A). An EHT was then induced in an indicated basic medium such as APEL2 (STEMCELL Technologies), with signaling factors: 20 ng/mL SCF for subsequent NK induction; additional 10 ng/mL FLT3L, and 20 ng/mL IL-7 for T cells; IL-3, TPO, and M-CSF in X-VIVO 15 (Lonza) for downstream monocyte differentiation. Emerging suspension cells were typically harvested at day 17 or 20.

### T and monocyte differentiation

Subsequent proof-of-concept induction of a T-cell fate was achieved by replating HPCs onto DLL4-coated 48-well plates in StemSpan SFEM II (STEMCELL Technologies) with SCF, FLT3L, and IL-7 (all at 50 ng/mL). Monocytes were differentiated from HPC intermediates using 20 ng/mL SCF and 50 ng/mL M-CSF in X-VIVO 15.

### NK-cell differentiation

Unless stated otherwise, day 17 cells were transferred to new 12-wells at 200 000/well in APEL2 medium with SCF, FLT3L, IL-7, and IL-15 at 20, 10, 20, and 10 ng/mL, respectively. Cultures were fed every 3-4 days applying 50% medium changes (2 mL total) until day 28 to then be repeatedly split in hPL-based medium (DF12 with 15% hPL—PL BioScience—1x Glutamax, and 250 µM 2-phospho-L-ascorbate, plus above factors).

### Nucleic acid sequencing

Whole-genome Illumina sequencing at ~50× coverage was outsourced (Alacris Theranostics). Associated GMP specifications are given in Supplementary Table S1. RNA-seq was carried out using standard Illumina or Nanopore sequencing on MinION flowcells (Oxford Nanopore; Supplementary Table S2).

### Gene editing

Knockouts were accomplished via nucleofection of RNP complexes (4D Nucleofector, Lonza, program CA167, solution P4, 2 RNPs per gene). The optimized knockin procedure was based on nucleofecting 3 µg of donor vector in addition to a single gRNA targeting *AAVS1*. All nucleic acid sequences are given in Supplementary Table S4. Controls for unspecific integration were vector + irrelevant gRNA. Optimization of KI efficiencies and survival included details like allowing cell recovery and exposing them to lower temperatures (32 °C) after nucleofection.

### PCR-based methods

Primer sequences are given in Supplementary Table S4. SYBR Green-based RT-qPCRs were carried out as described^13^ and results were expressed as % of *RPL37A* expression or relative to an indicated reference. Conventional PCRs and TaqMan assays followed standard procedures.

### Immunofluorescence-based methods

Flow cytometry and immunofluorescence microscopy were carried out according to standard procedures. Primary antibodies and controls are given in Supplementary Table S3.

### Statistical methods

Quantitative data were processed in MS Excel or GraphPad Prism. Replicates were biological, not technical throughout. Error bars in charts highlighting individual replicates indicate 95% CIs of the mean values, otherwise SDs. Statistical testing was done using 2-sided paired or unpaired *t*-tests against appropriate control samples. *P* values of <.05 (*) or <.01 (**) highlight statistically significant differences.

## Results

### GMP iPSC cell lines meet a comprehensive set of QC specifications

**Figure 1.**
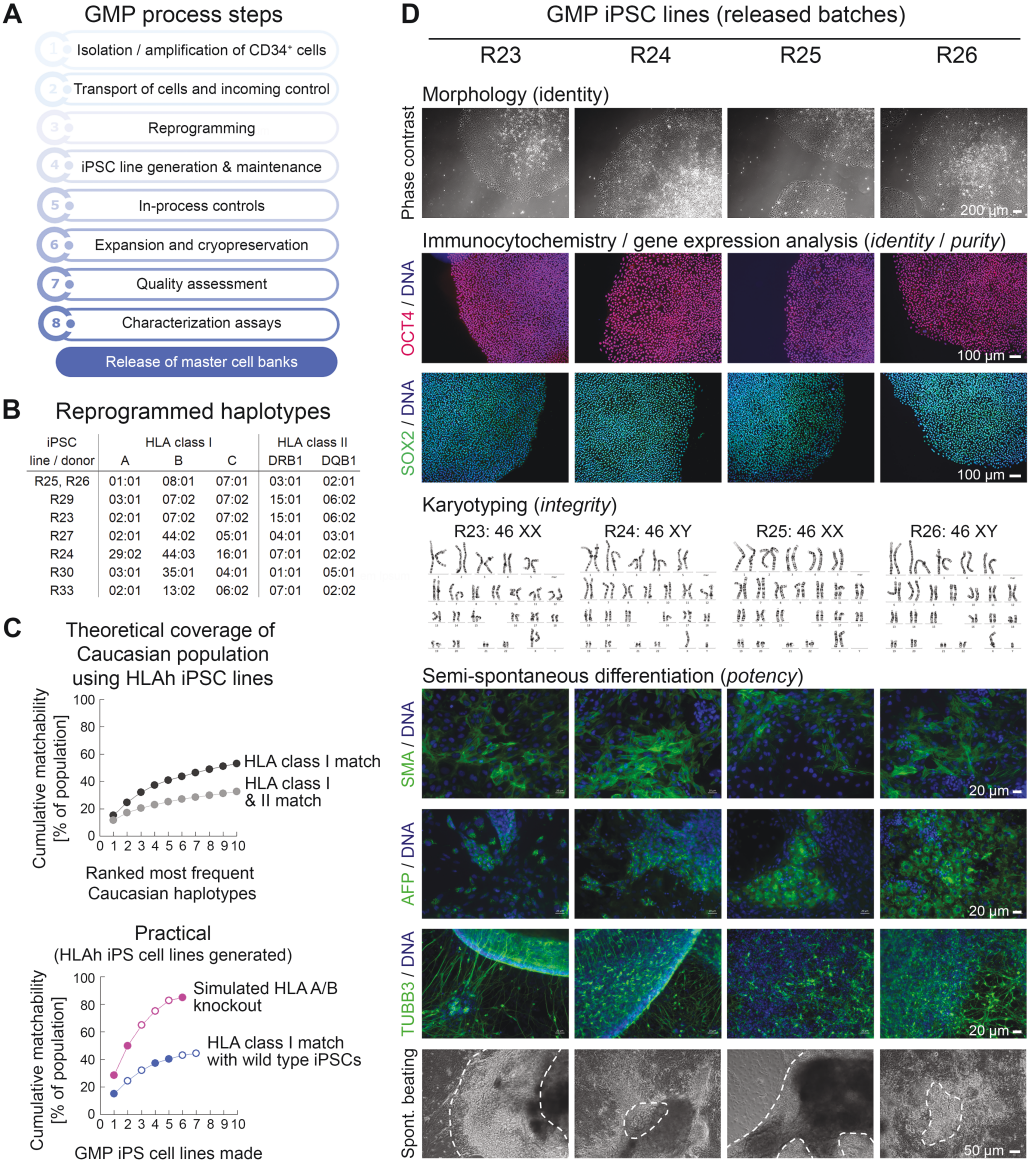
Reprogramming process and iPSC characterization. (A) Flowchart of iPSC manufacturing process with QC. (B) Top 7 most frequent Caucasian HLA haplotypes accessible for reprogramming from homozygous donors. R25 and R26 are iPSC lines derived from distinct individuals. (C) Probabilities of identifying matching patients with a given set of HLA-homozygous iPSC lines in the Caucasian population. Open circles in the bottom chart: reprogrammed iPSC lines with pending QC. Filled circles in same chart: released iPSC lines. Two released lines share the same (most frequent) HLA haplotype. (D) Exemplary characterization data of 4 reprogrammed iPSC lines showing the indicated features and markers.

As umbilical cord blood (CB) bears several advantages such as being a neonatal cell source and allowing selection of donors with HLA-homozygous genotypes, we established a GMP manufacturing workflow based on this starting material. CB units were selected based on informed reconsent and HLA haplotype frequency. CD34^+^ hematopoietic stem cells (HSCs) were isolated from these units and expanded ex vivo for 3 or 4 days in a separate process published elsewhere.^11^ The HSCs were then subjected to episomal reprogramming in a dedicated facility (Figure 1A). As further detailed in the Methods section, several iPSC lines were established and cryopreserved as seed stocks to prioritize one line for further expansion and cryobanking—after reprogramming vector elimination had been demonstrated via in-process controls.

HLA-homozygous iPSC lines have been established for 7 of the most frequent Caucasian haplotypes to date (Figure 1B). Herein, the validation batches, termed R23-26, covered ranks 1, 4, and 5 by frequency in the population (R25 and R26 share the same most frequent genotype). Collectively, the 7 lines would be able to match over 40% of the Caucasian population with regard to HLA class I, close to the optimum scenario (Figure 1C). As shown in the same figure (pink curve), a selective HLA-A and B double-knockout in these lines would further enhance their immune compatibility and extend it to other ethnicities.^16^

iPSC lines R23-26 as well as the starting material were extensively characterized based on the release specifications and additional assays (Supplementary Table S1). For instance, reprogramming vectors were required—and indeed found to be—undetectable by stringent TaqMan assays as well as by whole-genome sequencing in the 4 lines (Supplementary Figure S1A). The cells further showed normal hPSC-like morphology, gene expression profiles comparable to hES cell controls, normal karyotypes, and acquired ability to differentiate into derivatives of the 3 germ layers (Figure 1D, Supplementary Figure S1B-D).

In terms of genomic integrity, analysis of whole-genome sequencing data revealed a relatively low global mutation load as compared to adult tissue-derived iPSCs.^17^ This could be attributed to the neonatal starting material since that difference was mainly due to clonal mutations already present in the founder iPS cell (Supplementary Figure S1E). Otherwise, there were no functional mutations in a defined set of critical cancer-related genes in any of the lines, and no major copy number variations (Supplementary Table S1).

Although, hence, no abnormalities violating the GMP specifications were identified with any of the assays, the distribution of an R25 cell bank was put on hold as a precautionary measure. This was due to a suspected subchromosomal amplification of the 20q.11.21 region in a fraction of the cells—undetectable at the banking stage but detectable by ddPCR after long-term culture from independent vials. Duplication of chr 20q.11.21 is known to confer a selective advantage in continuously cultured human iPSCs whereas, notably, no genes of the Cancer Census Tier 1 set are contained in that region.^18,19^

In addition, line R24 turned out to be harder to handle than the other lines in terms of fully suppressing spontaneous differentiation, at least in our standard culture conditions using EDTA-based passaging. Therefore, subsequent efforts in devising improved manipulation workflows mainly employed R26 and R23, as well as a subsequently derived HLA-heterozygous line, R34.

### A defined induction cocktail accelerates efficient RPE differentiation from iPSCs

As a starting point for improving RPE differentiation from iPSCs, we first reproduced an attractive all-2D approach by Maruotti et al based solely on a treatment with small molecules.^20^ RPE cells were obtained from approximately 6 weeks onwards. However, additional supplementation of the known pro-RPE factor Activin A^21^ markedly accelerated the process, as evidenced by cell pigmentation and RPE marker gene expression (Supplementary Figure S2A and B). Nonetheless, as previously noted, the process was still accompanied by substantial cell death. We therefore replaced chetomin and high-dose nicotinamide treatment by defined inhibitors of the TGFβ, FGF, and BMP signaling pathways previously shown to promote accelerated neural induction.^14^

**Figure 2.**
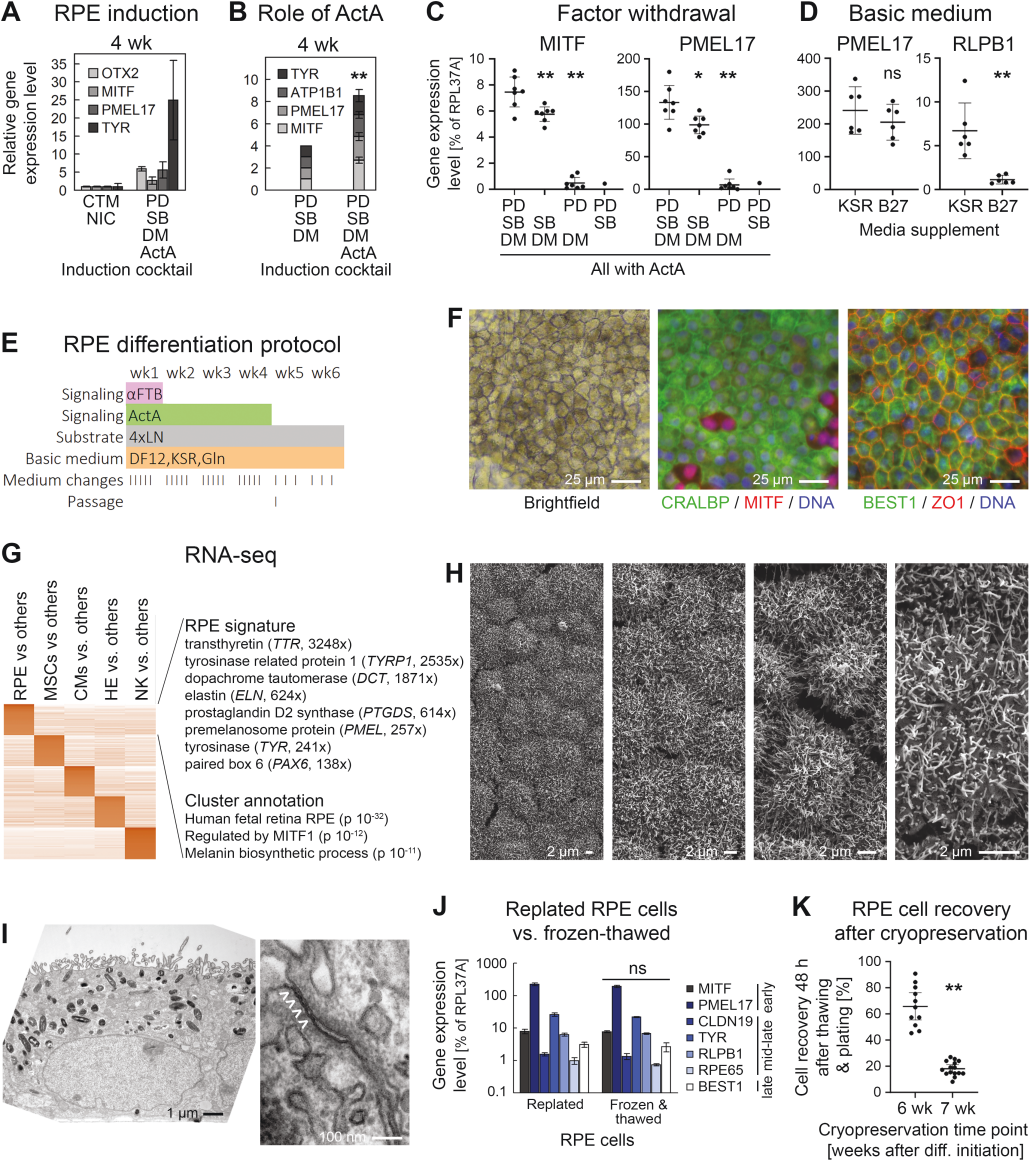
Directed all-2D RPE induction. (A) RT-qPCR analysis following RPE induction with 2 protocols (line R26, *n* = 2-6 per data point). PD: PD0325901 (αFGF), SB: SB431542 (αTGFβ), DM: Dorsomorphin (αBMP). (B) RT-qPCR data showing importance of Activin A supplementation in optimized induction cocktail (line R26, *n* = 5 for + ActA). (C) Analysis of individual contributions by 3 small molecules in the RPE induction cocktail (RT-qPCR data at 4 weeks). (D) Comparison of media supplements used in the new differentiation protocol. Low *RLPB1* expression was consistently observed with the B27 supplement. (E) Optimized RPE differentiation protocol. The FGF, TGFβ, and BMP pathways were (moderately) inhibited during the first week. Overlapping ActA treatment was productive for RPE induction. (F) Light and immunofluorescence analysis of maturated iPSC-RPE cells. A minority of (likely more immature) cells was MITF^high^/CRALBP-negative. (G) RNA-seq analysis of iPSC-RPE cells against other derivatives. Selected markers with ratios against other cell types as well as annotation terms of the RPE cluster are highlighted. (H) Scanning EM pictures at different magnifications reveal pronounced microvilli on all RPE cells. (I) Transmission EM analysis reveals apical-basal polarity with biased localization of melanosomes and nuclei (left) as well as tight junctions (right, marked with arrow heads). (J) Freeze-thawing preserves RPE marker expression (RT-qPCR analysis, *n* = 2). K, iPSC-RPE cells can efficiently be recovered from cryopreserved stocks by 6 but not 7 weeks of differentiation. All data in this figure are based on iPSC line R26.

The revised treatment regimen preserved cell survival and promoted an RPE fate more rapidly than the original protocol (Figure 2A) and each component of the cocktail indeed made a significant contribution (Figure 2B and C). Finally, we found that the complex and commonly used B27 media supplement had a negative effect on the expression of RPE maturation marker *RLPB1* (*CRALBP*), as compared to using Knockout Serum Replacement (Figure 2D). These and other optimizations resulted in an essentially new protocol depicted in Figure 2E.

Concentrations of the applied molecules and treatment times were optimized. Although few days of inhibiting the 3 pathways were sufficient to induce an RPE fate, a 1-week treatment diminished residual iPSC expression (Supplementary Figure S2C and D). This was confirmed in time course experiments which also revealed the kinetics of RPE marker gene induction in this protocol: *MITF* and *PMEL17* already reached close-to-maximum levels by 4 weeks, whereas maturation markers were more gradually induced, as expected (Supplementary Figure S2E). Hence, late markers CRALBP and BEST1 were readily detectable at protein level from 6 to 8 weeks and expression profiling confirmed a highly enriched RPE signature in these cultures (Figure 2F and G; Supplementary Table S2).

Functionally, the iPSC-RPE cells were homogeneously covered with microvilli as an important feature^22^ (Figure 2H), displayed apical-basal polarity with pronounced melanosomes and tight junctions (Figure 2I), and were, consequently, able to form tight layers with transepithelial electrical resistance (Supplementary Figure S2F). Moreover, the protocol was applicable to independent iPSC lines resulting in >95% pure RPE cells by 6 weeks, which supersedes any enrichment steps in a potential GMP process (Supplementary Figure S2G). Finally, as a pre-requisite for banking individual patient doses, frozen-thawed RPE cells were indistinguishable from replated ones by gene expression (Figure 2J). Along these lines, 6 week-old cells could interestingly be recovered from cryostocks at high efficiencies, whereas survival rates sharply declined thereafter (Figure 2K).

### Facilitated workflow yields highly proliferative iPSC-MSCs

**Figure 3.**
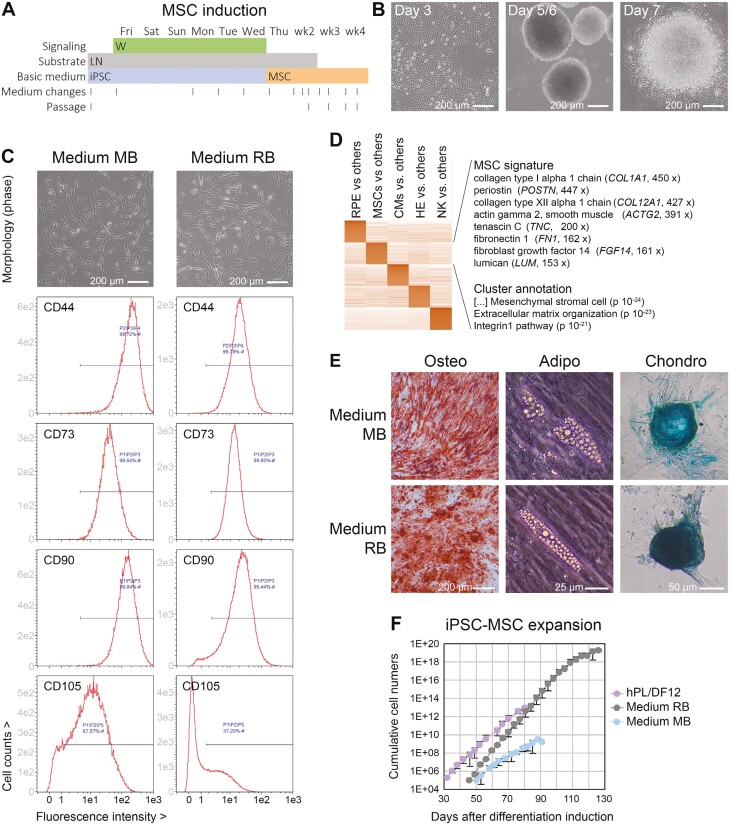
Single molecule-based induction of highly proliferative iPSC-MSCs. (A) Outline of protocol. W = stimulation of canonical WNT signaling. (B) Cellular morphology at different stages. Cells growing out from re-attached spheres had a fibroblastoid morphology from the beginning. (C) Morphology and surface marker expression of iPSC-MSCs derived from line R26 in 2 commercial MSC media. (D) RNA-seq analysis of R26 iPSC-MSCs against other iPSC-derived cell types showing a pronounced ECM signature. (E) Representative pictures/stainings indicating tri-lineage differentiation competence of R26 iPSC-MSCs. (F) Expansion potential of iPSC-MSCs in different media (*n* = 2-4 per data series).

Based on previous observations that high-dose BMP and/or WNT stimulation of iPSCs promotes mesenchymal cell fates,^13^ we performed optimization experiments resulting in a simple protocol for the induction of proliferative iPSC-MSCs. This is based on a 6-day treatment with the WNT agonist CHIR99021 at 4 µM, immediately followed by applying MSC expansion conditions (Figure 3A). Prolonged WNT stimulation resulted in a condensation reaction by day 5/6, whereas the subsequent switch to MSC media promoted re-attachment of the partially detached spheres and homogeneous outgrowth of fibroblastoid cells (Figure 3B).

Following an intermediate passage on laminin, the cells could thereafter be expanded on plain cell culture plastic, as expected for iPSC-MSCs, and they near-homogeneously expressed MSC markers CD44, CD73, CD90, and CD166. By contrast, CD105 expression was heterogeneous across 2 GMP-compatible MSC media used (Figure 3C and Supplementary Figure S3A). Nonetheless, there was no indication of competing non-MSC lineages by RNA-seq or flow cytometry analysis, regardless of the iPSC line of origin (Figure 3D and Supplementary Figure S3A). The cells also formed osteocytes, adipocytes, and chondrocytes in conventional assays and were hence termed iPSC-MSCs (Figure 3E).

Independent batches of the cells could be expanded long-term as a pre-requisite for upscaling. There were, however, striking differences between the MSC media used as well as between different donors: Commercially available medium RB (RoosterBio) outcompeted other MSC media and iPSC-MSCs from line R26 outcompeted other donors. Remarkably, a vast theoretical number of >10^18^ cells could readily be generated with the R26/medium RB combination, which would eliminate any need for immortalization (Figure 3F and Supplementary Figure S3B).

### Revised 3D cardiac induction protocol overcomes batch-to-batch variation

**Figure 4.**
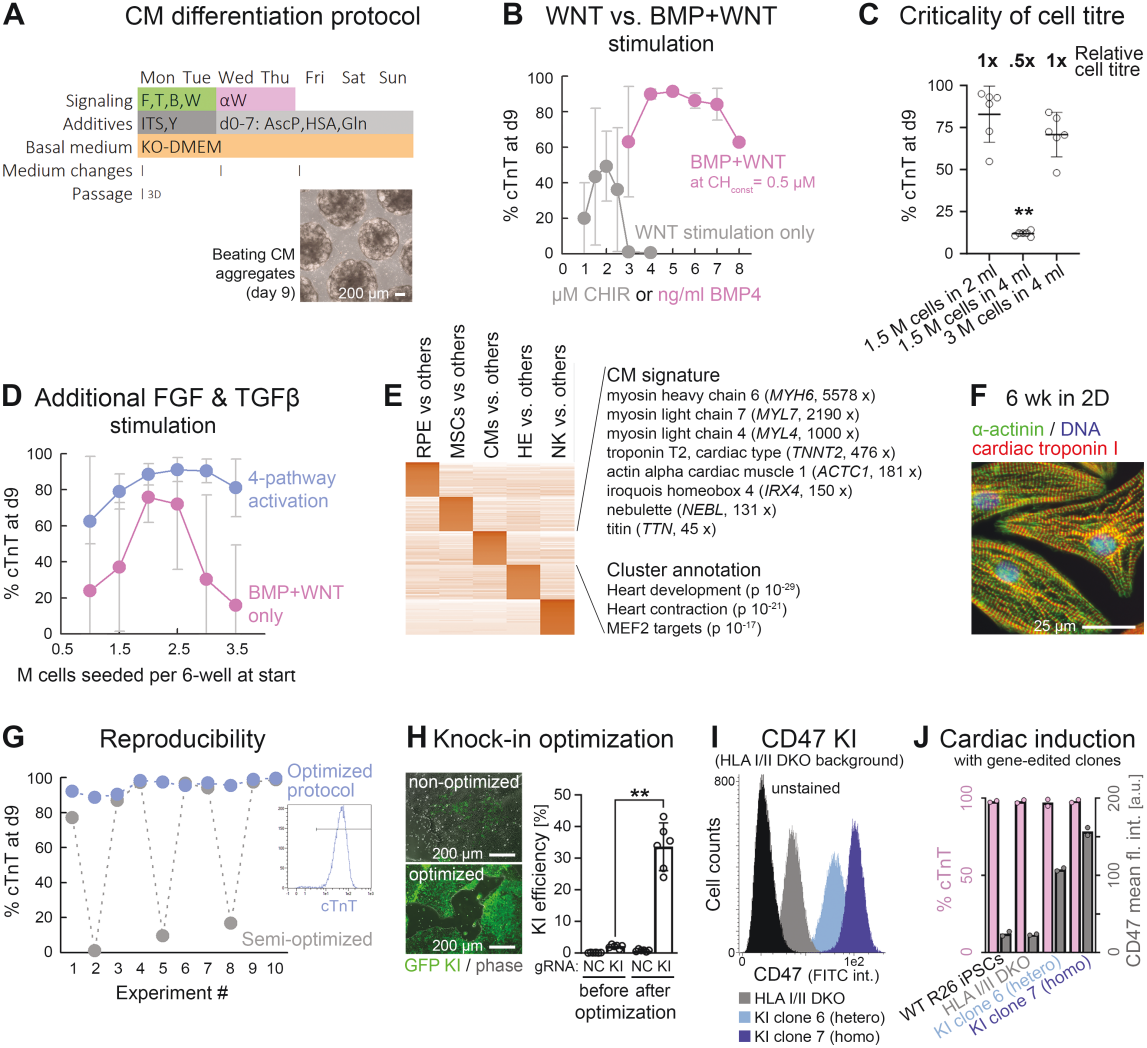
High-efficiency cardiac induction hallmarked by improved robustness. (A) Outline of optimized protocol based on initial co-stimulation of the FGF, TGFβ, BMP, and WNT pathways. Differentiation commenced upon—not after—EB formation. (B) BMP and WNT co-stimulation (without FGF/ TGFβ) enables superior CM differentiation as compared to WNT stimulation alone, in this protocol (*n* = 2-10 per data point). (C) Critical dependency of BMP + WNT protocol on cell titer (flow cytometry data). (D) Additional FGF and TGFβ pathway stimulation enhances protocol robustness with regard to cell titer dependency (*n* = 6-8—dropouts were scored as 0%). (E) Markers and annotation terms of cardiac differentiation cluster (RNA-seq analysis). (F) Immunostaining of iPSC-CMs maturated under adherent conditions. (G) Fully optimized procedure overcomes inter-experimental variation. Data shows flow cytometry data of 10 independent experiments conducted in a row. (H) Optimization of CRISPR-mediated knockin efficiencies in iPSCs using a GFP vector targeting the *AAVS1* locus. NC: Negative control using an irrelevant gRNA. (I) Representative flow cytometry plot showing negative controls as well as isolated hetero and homozygous knockin clones of a *CD47* transgene targeted to *AAVS1* on an HLA I/II deficient background. Note the correlation between signal and gene dosage. (J) Analysis of these iPSC lines after directed cardiac differentiation according to panel A. Data imply consistent differentiation after KO/KI editing and preserved gene dosage-dependent transgene expression.

WNT stimulation at the right dose can also induce cardiac mesoderm although this pathway may also cooperate with others in inducing that fate in a more robust manner.^13^ Interestingly, this co-stimulation approach has successfully been applied to 3D conditions, as well.^23^ We have therefore used this as a starting point for full optimization in a series of >100 experiments with regards to efficiency and consistency. The final protocol—which relies on differentiation induction *upon* not after 3D aggregate formation—is shown in Figure 4A.

Basic inducers of cardiogenic mesoderm initially were BMP4 and CHIR99021. Titration experiments revealed that the combined treatment did achieve high differentiation efficiencies as assessed by cardiac troponin C staining, whereas WNT activation alone in the present setup did not (Figure 4B). However, reproducing those promising outcomes based on BMP + WNT activation was difficult. Upon further experimentation, cell titer—regardless of the signaling factor concentrations—was confirmed as a critical process parameter.^24^ An exemplary outcome is shown in Figure 4C: Lowering the cell titer even by just 2-fold dramatically decreased cardiac differentiation efficiencies, despite keeping all other parameters constant.

We therefore hypothesized that accessory pathways such as FGF and TGFβ signaling may buffer the system by compensating for autocrine effects.^13^ Extending the co-stimulation cocktail by FGF2 and Activin A at an optimized dose enhanced cardiac differentiation efficiencies to some extent (Supplementary Figure S4A). Importantly, however, 4-pathway stimulation also enhanced the robustness of the protocol by reducing the criticality of cell titer as a seemingly independent process parameter (Figure 4D).

iPSC-cardiomyocytes (CMs) generated this way were initially characterized by a highly enriched but immature gene expression signature, as expected (Figure 4E). Over time, however, early myosin light and heavy chain-encoding genes (*MYL4*, *MYL7*, and *MYH6*) were replaced by late isoforms (*MYL2*, *MYH7*) and the cells acquired a more pronounced sarcomeric structure, in line with the idea of maturation toward a ventricular default fate (Supplementary Figure S4C and 4F).

We then challenged the protocol from a process consistency perspective by conducting 10 experiments in a row. Strikingly, there were still severe dropouts in some of the trials, which is unacceptable from a GMP standpoint (gray curve in Figure 4G). However, a few seemingly minor changes with regards to media change intervals and media additives like ascorbic acid finally established a truly robust methodology, at least at a small scale (Supplementary Figure S4D and blue curve in Figure 4G).

### Cardiac differentiation competence withstands 2 rounds of highly efficient gene editing

In parallel efforts, we sought to improve gene editing efficiencies in iPSCs in order to help enhance the application spectrum. As a paradigm, we used a hypoimmune approach similar to the compelling one by Deuse et al^9^ Hence, following optimization, we sought to functionally knock out HLA classes I and II by targeting cofactor *B2M* and the upstream regulator *RFXANK*, respectively. Homozygous double-knockout efficiencies were in the range of 50% (Supplementary Figure S4E) and, in isolated clones at the protein level, both HLA class I and II were indeed undetectable (Supplementary Figure S4F).

Achieving high knockin efficiencies—based on nucleofection of a targeting vector and a CRISPR RNP complex targeting the safe harbor locus *AAVS1*—was more challenging. Interestingly, however, a series of incremental improvements turned out to be additive in their effects. These modifications affected details about the nucleofection procedure such as program and cell numbers used, as well as handling steps thereafter, such as transiently exposing the cells to lower temperatures in order to favor homology-directed DNA repair over non-homologous end joining.^25^

In sum, these details enhanced specific knockin efficiencies from a few percent to over 30% on average (Figure 4H). When applying a semi-optimized version of the protocol to targeting a *CD47* transgene to the *AAVS1* locus, both hetero and homozygous knockin clones could readily be isolated (Figure 4I and Supplementary S4G-I). Interestingly, these 2 rounds of gene editing did not at all affect cardiac differentiation competence in any of the clones, which functionally confirmed the robustness of the differentiation procedure as well as the specificity of the gene editing steps (Figure 4J).

### Universal immune cell induction platform gives rise to multipotent precursor cells

In separate efforts, we were initially interested in optimizing differentiation along the endothelial lineage using a protocol by Patsch et al as starting point.^26^ By stimulating iPSCs with a combined high dose of BMP and WNT signaling followed by prolonged VEGF treatment according to Figure 5A (first 7 days), we obtained cultures that expressed the hemangioblast marker KDR, PECAM-1 (CD31), as well as other early endothelial genes at high levels (Figure 5A-C and Supplementary Figure S5A). After passaging, these cells acquired an endothelial morphology and tube formation capability (Supplementary Figure S5B and C), confirming that the day 7 cells had endothelial differentiation competence.

However, KDR is also a marker of early hematopoietic cells and strikingly, the day 7 cells co-expressed CD34, the prototype marker of hematopoietic stem cells (Figure 5B–C and Supplementary Figure S5A). We therefore hypothesized that the cells could also be turned into immune cells and termed them hemogenic endothelium (HE), as we provide further evidence for this claim. Interestingly, the day 7 cells formed clusters on an endothelial-like monolayer very much like embryonic HE clusters forming inside the dorsal aorta, the site of definitive hematopoiesis.^27^ In several media tested—defined or more undefined ones supplemented with SCF and FLT3L—the cells then clearly underwent an endothelial-to-hematopoietic transition (EHT) giving rise to live suspension cells (Figure 5D). This process was accompanied by an expression shift from a hybrid CD144^+^/CD34^+^/CD45^−^ HE signature to a CD144^−^/CD34^+^/CD45^+^ hematopoietic precursor cell (HPC) profile (Figure 5E).

As a proof of their identity, the suspension cells were subjected to myeloid-biased methylcellulose assays and indeed formed corresponding colonies at high efficiency, including erythroid ones (Figure 5F). As mentioned, different basic media supported the EHT process. Chemically defined medium devoid of serum-derived components, however, gave rise to a naïve CD34^+^/CD43^+^/CD117^+^/CD90^+^/CD38^−^ profile resembling that of bona fide hematopoietic stem cells, although we did not investigate their engraftment potential in vivo (Figure 5G). Similar results were obtained for 3 donors suggesting cell line independence of this HPC induction platform (Supplementary Figure S5D).

To provide further evidence of their multipotency, iPSC-HPCs were subjected to non-optimized downstream protocols. Using IL3 and M-CSF as additional cues,^28^ the HPCs could readily be differentiated into a monocyte/early macrophage fate marked by CD14, CD16, and others (Figure 5H). The process was interestingly accompanied by significant cell proliferation. Likewise, DLL4-coated surfaces successfully mediated the induction of T-cell progenitors, whereas the CD5 gate also contained a fraction of more mature CD4^+^/CD8^+^ cells and even T-cell receptor-expressing cells (Figure 5I). Despite being suboptimal in terms of efficiency, these initial protocols provide evidence that the underlying iPSC-HPC platform holds universal differentiation potential for both myeloid and lymphoid lineages.

### Media switch at precursor stage switch enables scalable generation of iPSC-NK cells

**Figure 6.**
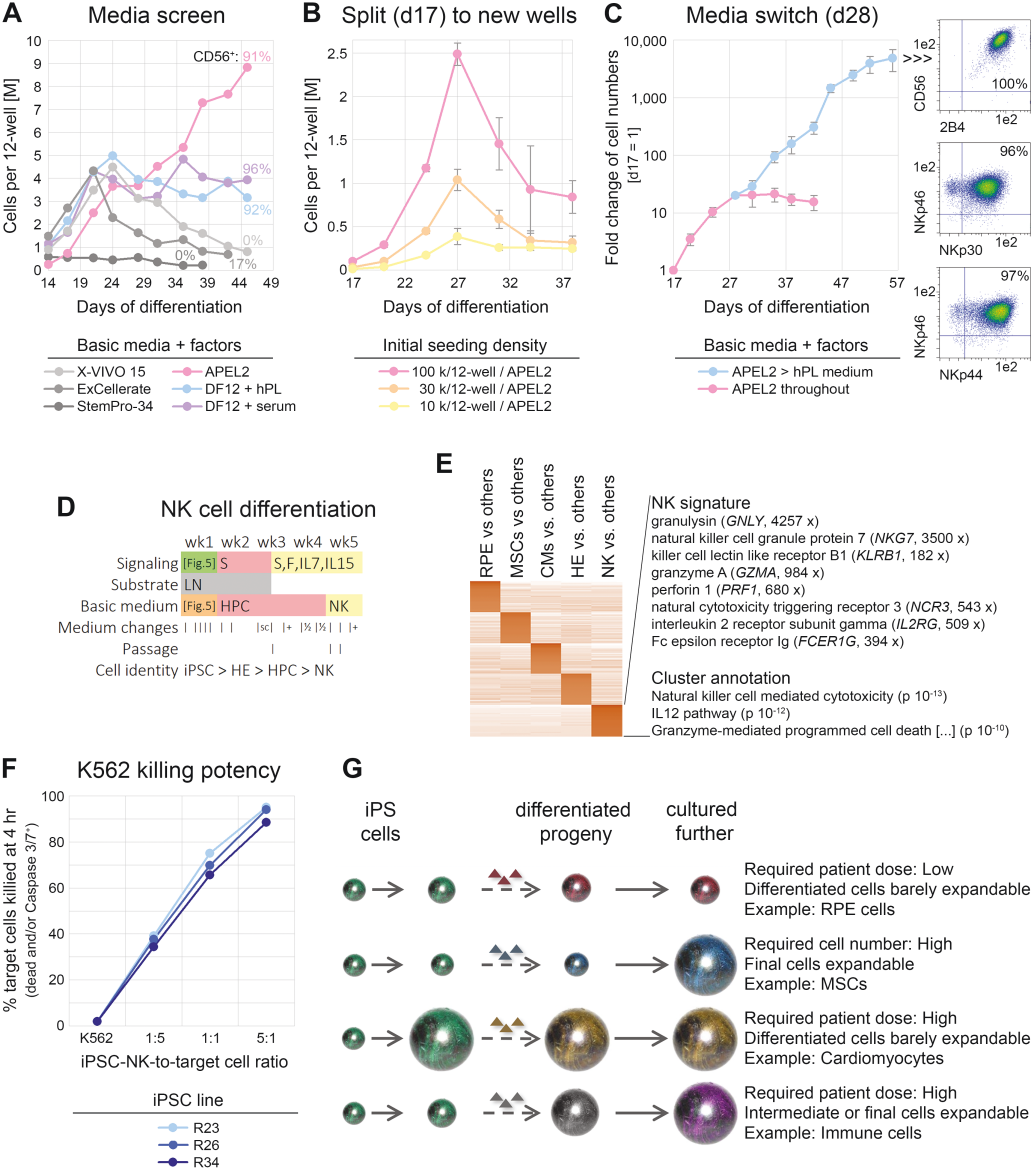
Basic media switch enables significant cell expansion upon NK-cell differentiation. (A) Screening of indicated basic media to support NK-cell differentiation and proliferation from iPSC-HPCs. All media were supplemented with SCF, FLT3L, IL-7, and IL-15. Cells remained in the original differentiation wells throughout (media switch on day 14). (B) Defined APEL2 medium with factors does not support longer-term cell expansion after transfer of HPCs to independent culture wells, regardless of cell density (*n* = 3 per data point). (C) Media switch at transition point between precursors and NK cells enables cell expansion upon differentiation (*n* = 6 similar conditions). Note the logarithmic scale. Right panel: Flow cytometric analysis at end of time course. (D) Resulting protocol with indicated media changes and added signaling molecules. S = SCF, F = FLT3L. Refer to Figure 5A for treatments over the first 7 days. (E) RNA-seq analysis highlighting NK-cell-specific cluster with selected marker genes and enrichment terms (also see Supplementary Table S2). (F) Killing assays using NK cells derived from 3 iPSC lines. (G) Illustration linking differentiation paradigms of the present study to context-dependent strategies for upscaling. Ball sizes are to reflect cell numbers.

Differentiation into another cell type of high relevance in immuno-oncology, natural killer (NK) cells, was developed more thoroughly. Using an established set of 4 signaling factors as a cue,^29^ we wondered which basic medium would best support NK differentiation in our system. A mini screen of distinct commercial as well as home-made basic media—all supplemented with the same signaling molecules—revealed striking differences. One sample based on chemically defined APEL medium^30^ stood out in terms of cell numbers and NK differentiation efficiencies marked by CD56 (Figure 6A). Another simple albeit undefined medium, DF12 supplemented with human platelet lysate (hPL) and L-ascorbate, also supported NK-cell induction but failed in expanding the cells in a sustained manner (blue curve in Figure 6A). Nonetheless, both these media robustly enabled NK-cell induction across independent iPSC lines based on the above HPC platform (Supplementary Figure S6A).

As these and other experiments were carried out on the original wells containing residual adherent cells, we sought to rule out indirect effects and investigate upscaling strategies after replating day 17 HPCs to new vessels. Strikingly, following a promising rise in cell numbers over approximately 10 days in APEL2 medium plus factors, the cultures began to deteriorate although many live cells could still be maintained and eventually acquired an NK fate (Figure 6B). These observations, together with data in Figure 6A, indicate that neither defined APEL2-based medium nor undefined alternatives alone supported substantial cell expansion *upon* NK-cell differentiation.

When investigating the kinetics of the loss of HPC and gain of NK-cell identity, we noticed with interest that the culture collapse in an APEL2-based medium at ~4 weeks coincided with a sharp HPC-to-NK transition point (Supplementary Figure S6B). We reasoned that the defined medium could probably support expansion of progenitor cells but failed to do the same after their transition into an early NK fate. Hence, we sought to evaluate whether undefined hPL medium could take over from that time point onwards. Remarkably, switching from APEL2 to an hPL-based medium at day 28 stimulated cell proliferation over several orders of magnitude while yielding pure NK cells as indicated by CD56 positivity and expression of functional markers (2B4, NKp30, NKp44, NKp46—Figure 6C and D).

Flow cytometry analyses suggested that ending the process with an undefined—yet fundamentally GMP-compatible—hPL-based medium may yield somewhat more mature cells characterized by elevated levels of CD38 and activation marker NKp44 (Supplementary Figure S6B). RNA-seq analysis of iPSC-NK cells derived in an hPL-based medium confirmed robust expression of various functional genes conferring cytotoxicity (Figure 6E). Indeed, the differentiated cells proved to be potent killers of K562 leukemia cells in that a 5:1 effector-to-target cell ratio caused a 90% death rate after just 4 hours of exposure (Figure 6F).

## Discussion

The purpose of this work was to reduce hurdles in iPSC-based therapy with regard to the starting material and downstream manipulation workflows. For instance, there is a general scarcity of off-the-shelf iPSC lines that meet GMP standards and donor eligibility requirements from a regulatory perspective, that allow commercial use from a legal/reconsent point of view, and that are easy to handle and manipulate from a technical standpoint.^31^ Several of the lines described and used here fulfill these criteria and shall prove useful in developing and executing therapeutic manufacturing processes. The disclosed QC specifications and additional characterization tend to exceed previously proposed guidelines^32^ and highlight some advantages associated with employing neonatal cells for reprogramming. Nonetheless, 2 lines (R24 and R25) that formally met all specifications turned out to be imperfect upon further investigation. This suggests that the release criteria might still need to be evolved in terms of enhancing their stringency and/or be extended by more specific assays for detecting subclonal chr 20q.11.21 amplifications.^18^

As indicated further above, the GMP framework imposes specific constraints and challenges to the manufacturing of iPSC-based products, which is difficult to anticipate from a basic research perspective. Moreover, different cell types of medical interest all require specific upscaling strategies depending on the inherent properties of the cells and the required patient dose as illustrated in Figure 6G. While gene editing and some cell types such as RPE do actually not require significant upscaling due to the low numbers of cells needed per patient (Figure 6G, first panel), the majority of cell types do. From a cost of goods and complexity standpoint, it thereby seems advantageous to exploit the inherent proliferation potential of the differentiated or differentiating cells whenever possible. For instance, the striking proliferation capacity of iPSC-MSCs from at least some iPSC lines shall allow for intermediate banking while still preserving sufficient expansion potential downstream (Figure 6G, second panel). It is currently unclear why the proliferation potential of R26-derived MSCs outcompeted other genetic backgrounds: In its ground state, R26 iPSCs grow well and a little bit faster than R23 cells but not significantly faster than additional lines investigated (R34 and data not shown).

By contrast, iPSC-derived cardiomyocytes expand rather poorly such that upscaling at the iPSC stage seems inevitable (Figure 6G, third panel). Indeed, encouraging preliminary data suggests that our 4 factor-based induction protocol may be scalable (data not shown). Finally, with regard to immune cells, it may be strategic to upscale at a precursor stage and avoid exhaustion of the final product (Figure 6G, fourth panel). To the best of our knowledge, our approach to expanding iPSC-NK cells *upon* differentiation is the first disclosed one of its kind.

Overall, these new workflows are all characterized by advantageous hallmarks. For instance, GMP-friendly improvements enhanced gene knockin efficiencies in an additive manner, overall avoiding the need for selection or viral transduction strategies.^33^ The RPE and iPSC-MSC protocols are particularly simple, which resulted from efficient induction combined with selective culture conditions. Moreover, our cardiac induction platform appears to overcome—at least on a moderate scale—the pressing issue of batch-to-batch inconsistency. Finally, by extrapolation, substantial numbers of iPSC-NK cells could be generated from universal iPSC-derived HPCs without the need for inactivated cancer cells as feeders.^8^ In sum, these critical improvements will allow for applying these workflows under classified conditions where process consistency is key to avoid deviations and risks.

As a side note, the optimized manipulation protocols described here may also serve as a basis for studying mechanisms of human cell fate conversions from a mechanistic point of view. As an example, the fascinating EHT process giving rise to the first definitive hematopoietic cells in the human body appears to be much under-investigated owing to its inaccessibility in vivo. Combining loss and gain-of-function studies using optimized gene manipulation with the directed differentiation platform of the present study would present a powerful approach in this regard.

## Conclusions

Future work ahead will have to demonstrate the scalability of these protocols as required. While adherent cell types such iPSC-derived RPE cells or MSCs may conveniently be expanded on 2D surfaces which merely need to be enlarged, scaling processes in 3D may be more challenging. This is because additional parameters such as sheer force come into play and procedures for cell processing need to be adapted to the culture format. A pre-requisite for cardiac induction at a large scale will also be to first expand the iPSCs accordingly. Although some systems for 3D expansion of iPSCs have been developed, we are not aware of a study demonstrating consistent and highly efficient cardiac differentiation directly from such preparations in a scalable manner. Furthermore, although the manipulation workflows of the present study address and solve some challenges for therapeutic applications, others remain. For example, cell line variability in terms of iPSC differentiation efficiency or yield is not fully overcome by all our protocols, which reinforces the need to create panels of GMP cell lines to screen and ultimately choose from.

## Supplementary material

Supplementary material is available at *Stem Cells Translational Medicine* online.

szae047_suppl_Supplementary_Figures

szae047_suppl_Supplementary_Table_S1

szae047_suppl_Supplementary_Table_S2

szae047_suppl_Supplementary_Table_S3

szae047_suppl_Supplementary_Table_S4

## Data Availability

Non-GMP evaluation stocks of the GMP iPSC lines are available based on a material transfer agreement. Genomic sequences of the iPSCs may not be disclosed due to data protection clause in the donor reconsent. All other relevant data are available in the main article or supplementary information.
