# Supplementary material for: GMP-compliant iPS cell lines show widespread plasticity in a new set of differentiation workflows for cell replacement and cancer immunotherapy
Source: Stem Cells Transl Med. 2024 Jul 23;13(9):898–911. doi: 10.1093/stcltm/szae047 (PMC11386223; doi:10.1093/stcltm/szae047)
Supplement: szae047_suppl_Supplementary_Figures [file szae047_suppl_supplementary_figures.pdf]

SUPPLEMENTARY METHODS

Study Design

Cellular reprogramming was performed in a dedicated clean room facility in a class A-in-B setting. Its hardware, environmental monitoring system, the production process and in-process controls, storage and cryopreservation setup, hygiene concept, as well as analytical facilities and methods were assessed in a series of inspections by the local government (Bezirksregierung Düsseldorf) together with the Paul Ehrlich Institute. This resulted in a global GMP certification of the facility and a manufacturing authorization for the iPSC reprogramming process.

Accordingly, instruments and suppliers were formally qualified, measuring devices maintained and calibrated, analytical methods validated, critical raw materials tested, clean room personnel formally trained and qualified, and, following aseptic simulations, the overall process validated based on 4 batches produced in a consecutive manner. Batch records and process documentation templates were designed to also meet several FDA-specific GMP requirements. QC testing which implied matrix validations for safety-related assays is summarized in Table S1.

Reprogramming Process and QC

Logistically, the iPSC manufacturing process is preceded by the isolation of $CD34^+$ cells from cord blood units donated and consented for clinical use. CB units as well as blood samples of the mothers were safety-tested and genotyped according to clinical standards, which also included HLA typing. Documentation of the starting material further included filled questionnaires related to the families' medial history as well as an informed reconsent devised by a medical law firm and approved by the University Hospital of Düsseldorf (Germany). The donor reconsent enables CB conversion to iPSCs for therapeutic applications including commercial use. Part I of the GMP process leading to purified and transiently expanded $CD34^+$ cells as starting material for reprogramming has been described elsewhere.[11]

Following validated transport to the reprogramming facility, up to 1 M $CD34^+$ cells were subjected to episomal reprogramming adopting plasmid backbones by Okita et al.[12] The specific cocktail of factors is considered proprietary but contains, as an example, the *hsa-miR-302/367* cluster and avoids, for safety reasons, the dominant negative *TP53* insert. Vectors were synthesized at Eurofins Genomics (Ebersberg, Germany), followed by amplification/purification and extensive QC testing at PlasmidFactory (Bielefeld, Germany). Nucleofected $CD34^+$ cells were cultured in $CD34^+$-supportive medium on 6-well dishes coated with cell therapy-grade Laminin-521 (BioLamina # CT521-0501) at various dilutions favoring subsequent isolation of clonal iPSC colonies. $CD34^+$ medium was progressively replaced by StemMACS™ iPS-Brew GMP medium (Milteny Biotec # 170-076-317, 170-076-318, 170-076-166) within one week - iPSC media addition (without TGFβ1) on days 2 and 4, complete exchange (with TGFβ1) at day 7. Several isolated emerging iPSC-like colonies from the lowest possible dilution were manually picked in grade A to establish candidate lines. These were propagated using EDTA-based splitting as clusters for few passages to allow for vector elimination and near-complete reprogramming. Several of these lines were then cryopreserved as seed stocks and tested for hPSC-like gene expression as well as vector status, based on established in-process controls. One selected clone was further maintained under clean room conditions while continuously monitoring vector elimination once per week. Once complete vector clearance was demonstrated in 2 consecutive passages using stringent TaqMan assays over 40 cycles - typically below P10 - the iPSC line was expanded and ultimately cryopreserved (under quarantine conditions)

as a prospective GMP cell bank. Additional in-process control samples were taken immediately before the cells were cryopreserved in fully closed tubes (CBS # 022251).

QC specifications are provided in Table S1. The corresponding assays were formally validated and probed immediately before cryopreservation as mentioned or, alternatively, after reconstitution of the cells to monitor more general / stable characteristics of a given iPSC line. Assays outsourced to qualified suppliers comprised testing for safety and genome integrity (sterility, endotoxin, mycoplasma, viral contaminants, karyotyping, whole-genome sequencing), and identity (STR analysis). In-house assays on recovered cell material further addressed aspects of product integrity (recovery after thawing), identity / purity (hPSC marker expression at RNA and protein levels), impurity (vector elimination assay), and potency (semi-spontaneous differentiation assay). 2 human ES lines served to validate outcomes in some of these assays and to define quantitative ranges for assessing hPSC-like gene expression. Global method descriptions applying both to GMP and R&D conditions are provided further below. Antibodies and primer sequences are in Tables S2 and S3, respectively.

More specifically, cell recovery was assessed using Neubauer chambers with Trypan Blue exclusion. Vector elimination in QC was monitored using two independent TaqMan assays against the backbone of the reprogramming vectors with release specifications requiring no signal with both assays performed in triplicate. Full elimination of any vector sequence was also confirmed by whole-genome sequencing of the iPSCs. For assessing acquired pluripotency, the iPSCs were cultured as embryoid bodies under biased conditions either favoring mesendoderm induction based on WNT and TGFβ activation followed by WNT inhibition - or favoring neuroectoderm induction promoted by TGFβ and BMP signaling inhibition. Outgrowths from plated EBs were separately analyzed for the two conditions, to also confirm physiological responsiveness of the iPSCs to defined signaling stimuli.

Beyond the release assays underlying the QC specifications listed in Table S1, the validation batches were additionally characterized by RNA-sequencing to confirm equivalence to hESCs at transcriptome level (Alacris Theranostics, Berlin, Germany), by Illumina SNP arrays to reveal larger CNVs and compare to WGS results (CeGaT, Tübingen, Germany), and by ddPCR to assess CNVs specifically in critical regions across the genome (iCS-digital™ PSC 24-probe test - Stem Genomics, Montpellier, France).

iPSC differentiation

Prior to differentiation, iPS cells were maintained in StemMACS™ iPS-Brew XF medium (Miltenyi # 130-104-368, or equivalent GMP product) on 0.15 µg/cm² iMatrix-511 (Amsbio # 892011, equivalent GMP product available). iPSCs under R&D conditions were routinely replated twice per week in the presence of 10 µM Y-27632 (Tocris # 1254, alternative GMP product available) following 20-25 min of digestion with Accutase® (Merck # A6964, GMP equivalent available) containing the molecule. Cell culture conditions were 37°C / 5% CO_2 at saturated humidity throughout. Optionally, antibiotics were added to cell cultures under R&D conditions (ThermoFisher # 15070063).

RPE differentiation. A published protocol was reproduced using 25 nM chetomin (SigmaAldrich # C9623) and 10 mM nicotinamide (SigmaAldrich # N3376) and B27™ (ThermoFisher # 17504044 - with VitA) as basic media supplement.[20] The RPE differentiation protocol is depicted in Fig. 2E and commenced with confluent momolayer cultures of undifferentiated iPSCs on 0.6 µg iMatrix-511 per cm² (4x compared to standard culture condition). Medium was switched to basic RPE medium - 15%

(v/v) Knockout™ Serum Relacement in DMEM/F12 with 1x Glutamax (ThermoFisher # 10828028/12618013, 21331020/A1370801, and 35050038) - with specific factors. These were R&D-grade PD0325901 (AxonMedchem # Axon 1408, 1 µM), 5-10 µM SB431542 (Merck # 616461), 0.25 µM Dorsomorphin (Tocris # 3093), and 25 ng/ml Activin A (R&D Systems # 338-AC) - or equivalent GMP products - during the first week, and Activin A alone for the subsequent 3 weeks. Media volumes per 6-well were 4 ml for daily changes and 8 ml over weekends. RPE cells were replated using 5-10 min incubation with 10x TrypLE Select (ThermoFisher # A1217701) and cryopreserved in basic RPE medium with 10% CryoSure-DMSO (Wako # WAK-DMSO).

Differentiation into MSC-like cells. According to Fig. 3A, iPSCs were reseeded at 30,000 cells per 6-well under iPSC maintenance conditions. Over the next days, while applying the indicated media changes at 3-4 ml per well, the cells were treated with 4 µM CHIR99021 (Axon Medchem # Axon # 1386, alternative GMP products available) for 6 days in iPS Brew medium. Due to partial detachment of condensed balls of MSC progenitors towards the end of the treatment, the final medium changes were carried out with care. MSC-supportive media were used hence after, which resulted in firm attachment again and promoted outgrowth of MSC-like cells from the condensed spheres. MSC media were medium RB (RoosterNourish™-MSC-XF, RoosterBio # KT-016), medium MB (StemMACS™ MSC XF, Miltenyi Biotec # 130-104-182), or DMEM/F12 with 10% human platelet lysate (PL BioScience # PE31011). Typically 1.5 wk later, bulk cultures with MSC-like cells were replated at onto iMatrix-511 again using 1x Tryple Select (ThermoFisher # A1285901, with optional EDTA pretreatment in case of using medium MB). Subsequently, the cells could be maintained on plain tissue culture plastic while split ratios could be progressively increased depending on MSC medium and iPSC line of origin. In medium RB, iPSC-MSCs were typically split every 3-5 days without intermediate medium changes. Growth curves were recorded based on numbers of harvested and reseeded cells upon passaging. iPSC-MSCs were differentiated for 4 weeks using adipogenesis, osteogenesis, and chondrogenesis differentiation kits (ThermoFisher # A1007001, A1007101, and A1007201, respectively) following instructions by the manufacturer. Differentiated cells were (optionally) stained with 0.3% (v/v) Oil Red O (adipo / Merck # O1391 / 20 min), 40 mM Alzarin Red (osteo / Merck # TMS-008 / 2-3 min), or 1% (w/v) Alcian Blue (chondro / Merck # TMS-010 / 45 min) solutions following fixation with formaldehyde and washing with PBS, to be rinsed thereafter with distilled water.

Cardiac differentiation. Various parameters of the cardiac differentiation protocol of Fig. 4A were optimized under 3D conditions in standard 6-well tissue culture plates which were incubated on orbital or wave-type rockers in cell culture incubators. Prior to differentiation, iPSCs were grown to confluency in iPS Brew medium on iMatrix-511 and harvested by a 20-25 min digest with Accutase containing Y-27632. Washed cells were resuspended at 1.25 M/ml (2 ml per well) in basic cardiac differentiation medium (Knockout™ DMEM, ThermoFisher # 10829018 / 0.1% (w/v) HSA, Biological Industries # 05-720-1B / 250 µM 2-phospho-L-ascorbate / 2 mM L-glutamine), with additional factors (1x ITS, BD # 354351 / 3-5 ng/ml BMP4, R&D Systems # 314-BP / 1 µM CHIR99021, Axon Medchem # 1386 / 7.5 ng/ml FGF2, Peprotech # 100-18B / 7.5 ng/ml Activin A, R&D Systems # 338-AC-500 / 10 µM Y-27632 - all available in GMP-grade). 2 days later, culture conditions were switched to WNT inhibition using C-59 (Tocris # 5148 - 0.5 µM final in basic CM differentiation medium). Another 2 days later, as well as on d7, the culture medium was replaced by basic differentiation medium without factors. Knockout™ DMEM could optionally be replaced by other base media. With regards to Figure 4G, the improvement in protocol robustness was mainly achieved by only performing an incomplete (25%) medium exchange on day 2 (patent pending).

Hematopoietic induction. For the induction of HSC-like cells, iPSCs were reseeded under standard conditions into 12-well plates, typically at 200 k/well. Hemangioblast-like mesoderm was induced the next day by switching to 3 ml/well serum-free StemPro™-34 medium (ThermoFischer # 10639011) with 25 ng/ml BMP4 (Peprotech # 120-05ET) and 8 µM CHIR99021 (StemCell Technologies # 72054) for 3 days (Figure 5A). HE-like cells were then induced over the next 4 days in StemPro™-34 with 200 ng/ml VEGFA (StemCell Technologies # 78073) while applying daily media changes. Optionally, 2 µM Forskolin (Abcam # ab120058) or 10 µM SB431542 were selectively added for subsequent endothelial or hematopoietic differentiation, respectively.

For subsequent differentiation along the endothelial lineage, d7 cells were dissociated into single cells using Accutase and replated onto fibronectin-coated dishes (Corning # 356008, 2 µg/cm²) in StemPro-34 (or other with 100 ng/ml VEGFA, to be propagated further under these conditions. Tube formation assays were carried out by solidifying 200 µl/well Matrigel® (SigmaAldrich # CLS354234) in 96-well plates in the incubator, followed by the addition and over-night incubation of 10,000 EPCs per well resuspended in 150 µl StemPro34 with VEGFA.

For subsequent differentiation along the hematopoietic lineage, d7 cells were fed according to Fig. 5A (volume: 1 ml per 12-well) until the hemogenic clusters had dispersed into suspension cells. Different basic media and cocktails of added factors fundamentally supported the EHT process, albeit at varying efficiencies and yields. APEL2 medium (StemCell Technologies # 05270) was preferred for subsequent T and NK cell induction, whereas X-VIVO™ 15 (Lonza # BE02-053) was preferred for differentiation into monocytes. Optional or mandatory factors added during EHT for subsequent T and NK cell differentiation were SCF (R&D Systems # 7466-SC) at 20 ng/ml, FLT3L (R&D Systems # 308-FKE) at 10 ng/ml, and IL-7 (R&D Systems # 207-IL) at 20 ng/ml. For subsequent differentiation into monocytes, lymphoid-specific IL-7 in this cocktail was replaced by IL-3 (until d14, R&D Systems # 203-IL) at 5 ng/ml, TPO (Peprotech # 300-18) at 10 ng/ml, and M-CSF (R&D Systems # 216-MC) at 20 ng/ml. Media change intervals were as depicted in Fig. 5A. Emerging suspension cells were typically harvested at d17 or 20, unless stated otherwise. Then, these HPCs were either cryopreserved as mini banks or used for further differentiation into one of the three cell types:

T cell differentiation (non-optimized). For T cell differentiation beyond the d17 HSC-like stage, $CD34^+/CD43^+$ suspension cells were plated at 4000/well onto 48-well plates coated with DLL4 (9 µg/cm², Sino Biologicals # 10171-H02H) containing 300 µl StemSpan™ SFEM II (StemCell Technologies # 09605) with SCF, FLT3L, and IL-7 (all at 50 ng/ml). Partial media changes (200 µl/well) were performed every 3-4 days until d42 while transferring the cells to freshly coated wells once per week.

Monocyte differentiation (non-optimized). HSC-like cells were transferred to new wells in the aforementioned medium (1 ml/well). 1 ml of fresh medium was added on d17. Differentiation beyond d17 proceeded by performing 50% medium changes using X-VIVO 15 with SCF at 20 ng/ml and M-CSF at 50 ng/ml until d40.

NK cell differentiation. Differentiation beyond d17 was initially conducted in the original wells or, after optimization, in independent wells. In the original wells, a 2 ml/well spin-change preserving the suspension cells was conducted on d20. Thereafter, 50% media changes were performed every 3-4 days. Basic media were defined APEL2 medium or 15% hPL in DF12 with 1x Glutamax and 250

µM 2-phospho-L-ascorbate containing, in both cases, SCF, FLT3L, IL-7, and IL-15 (R&D Systems # 247-ILB) at 20, 10, 20, and 10 ng/ml, respectively. Alternative initial media included ExCellerate™ (R&D Systems # CCM030). When transferring d17 cells to new wells for partially optimized expansion during differentiation, the process first proceeded in 1 ml APEL2 medium with these same signaling factors and 200,000 transferred cells per 12-well. Medium was topped up to 2 ml on d20, followed by 50% medium changes until d28. Then, cultures were split 1:2 in the original medium and topped up to 2 ml/well with the above hPL-based one. From d31 onwards, the cultures were split once per week by transferring 500,000 cells to new 12-wells with 1 ml hPL-based medium, followed by topping up to 2 ml 2 days later, followed by a 50% medium change another 2 days later. Samples for cell counting and profiling were taken at the indicated time points.

Nucleic Acid Sequencing

Genomic samples of iPSCs and their starting material were prepared using DNA isolation kits (Qiagen # 69504) and subjected to whole-genome Illumina sequencing at ~50x coverage (Alacris Theranostics). Besides using the data for confirming the HLA haplotypes of the cells as well as the absence of any reprogramming vector sequence, the genomes were mined for structural variations and for mutations in iPSCs against the starting material. Details of the algorithms are proprietary but fundamentally, larger genomic deletions and duplications were scored requiring both (i) statistically significant alterations of the sequencing read density across a given candidate region and (ii) identification of paired-end reads unequivocally spanning the corresponding chromosomal break points. Results were found to be in agreement with Illumina SNP array data generated in parallel. Small mutations in iPSCs were scored requiring 3 or more reads displaying the same lesion, to exclude false positive hits due to sequencing errors in a 3 billion bp genome. GMP release criteria prohibited newly acquired CNVs >5 Mb as well as functional mutations in a set of 21 critical cancer-related genes including, for instance, *TP53* (Table S1). Other CNVs, mutations in other genes of the Cancer Gene Census Tier 1 panel,[19] or genomic lesions in the starting material were addressed through risk analyses. A meta-analysis comparing the global mutation load in adult tissue-derived iPSCs vs. cord blood-derived ones described here employed data and the same bioinformatics tools as in the reference study.[17] As in D'Antonio et al., clonal SNVs were defined as affecting around 50% of the reads whereas subclonal SNVs marked a second well-separated peak at the lower end of the mutation frequency spectrum. Similar to the reference paper, iPSC-specific mutations were unequivocally identified by comparison against the bulk starting material - cord blood in this present study.

Conventional RNA sequencing of iPSC lines R24-26 against their respective starting material and hESCs was carried out at Alacris Theranostics using Illumina instrumentation and standard bioinformatics analysis tools. RNA-seq of undifferentiated R26 iPSCs against differentiated progeny was performed using Oxford Nanopore MinION R9 flowcells (Oxford Nanopore # FLO-MIN106D) on a corresponding MinION device. RNA was isolated from undifferentiated R26 iPSCs as well as differentiated progeny cultured and processed as described in above sections using a Machery Nagel NucleoSpin RNA kit (# 740955.250). Library preparation was carried out using a PCR-cDNA sequencing barcoding kit as instructed by the manufacturer (Oxford Nanopore # SQK-PCB109) together with recommended accessory reagents. PCR-amplified cDNA (14 cycles, 3 min extension) was quantified using Qubit 1x dsDNA HS assays (Thermo # Q33231). Basecalling was performed using Guppy (Oxford Nanopore), on a Linux desktop computer. Mapping to known genes and scoring of sequencing reads was carried out using Dusselpore executed locally on a Docker Container.[34] Extracted counts per transcript were processed using basic functions in MS Excel (Data file S1).

Enrichment analysis in marker gene sets based on Ensembl IDs as input was conducted via Metascape.[35]

Gene Editing

DNA vectors and gRNA sequences are given in Table S4. Knock-in vectors were synthesized at GenScript (Piscataway, NJ) and provided in a standard cloning vector, amplified, and finally reconstituted in buffer EB (Qiagen # 19086) at 1-1.5 µg/µl. Synthetic sgRNAs (TE buffer 100 µM in TE buffer) and Cas9 (61 µM) or Cas12a nuclease (Cpf1, 63 µM) were from Integrated DNA Technologies (IDT # 1081061 or 10001273, respectively). Both enzymes showed similar performance overall. Gene knockouts were accomplished by excising defined DNA fragments with 2 separate gRNAs in gene bodies.

RNP complexes were prepared shortly before nucleofection by mixing 2.46 µl Cas9 or 2.42 Cas12a enzyme with 4.5 µl gRNA (single gRNA for KI into *AAVS1* or mix of 4 in case of double knockouts) followed by a 15-20 min incubation at RT. The HLA I/II double knockout with additional knock-ins were generated in a sequential manner. For nucleofection using a P4 Lonza 4D nucleofector kit (# V4XP-4024), iPSCs were harvested from a semiconfluent state by a 20-25 min Accutase incubation followed by centrifugation, resuspension in PBS containing 10 µM Y-27632 or 1x CloneR2 (StemCell Technologies # 100-0691), and cell titer quantification. 3 M or all harvested cells were pelleted again, resuspended in 100 µl P4 solution, mixed with 9 µg KI vector or RNP complexes (as above), respectively, to then apply program CA167. Cells were allowed to recover by adding 400 µl base medium with 10 µM Y-27632 or 1x CloneR2, followed by incubation at 37°C for 10 min. All nucleofected iPSCs were then plated out on an iMatrix-511-coated 6-well in iPS Brew medium containing 10 µM Y-27632 or 1x CloneR2. A key twist for achieving high cell survival and knock-in efficiencies was to deliver donor plasmid and gRNA complexes in a sequential manner (~24 hr apart - patent pending).

Up to one week later, when the survived iPSCs had resumed exponential growth, cultures were replated again, at a dilution compatible with clonal colony picking after 1-2 weeks. Alternatively, the dissociated cells could be clonally dispensed into iMatrix-511-coated 96-well plates using a DispenCell device (SEED Biosciences). Bulk cultures were analyzed immediately before clonal reseeding. Isolated candidate clones were further propagated to sufficient numbers to then be screened based. GFP expression, live cell staining using a CD47 antibody (Table S3), and/or PCR using primers in Table S4. For confirming HLA I/II deficiency in double-knockout cells, the iPSCs (for HLA I) or iPSC-derived endothelial precursors (for HLA II) were pre-treated with 100 ng/ml interferon-γ (Peprotech # 300-02) for 3 or 7 days, respectively, to induce sufficient HLA amounts in positive control cells. Optimization of KI efficiencies and associated cell survival rates included details about the nucleofection procedure such as allowing recovery and transiently exposing the cells to lower temperature (32°C) post nucleofection or using the CloneR2 reagent instead of Y-27632 after seeding bulk cultures.

PCR-based Methods

Primer sequences are given in Table S4. Conventional PCRs were carried out using Q5® High-Fidelity DNA Polymerase (NEB # M0491). cDNA was prepared using 1-2 µg total RNA and Promega # M1701 M-MLV reverse transcriptase with 0.5 µg oligo-sT_{15} priming in 25 µl reactions for 1 hr at 42°C. Otherwise, SYBR Green-based RT-qPCRs were carried out as described,[13] using prevalidated assays on a BioRad CFX96 or ABI QuantStudio 3 device. TaqMan assays were executed based on DNA

templates with TaqMan™ Universal PCR Master Mix (ThermoFisher # 4304437). RT-qPCR results were either expressed at an absolute scale as % of *RPL37A* expression (given standardized rules for primer design across genes and careful testing of primer pairs), or relative to an indicated control sample, or relative to the sample with highest gene expression in a given set of samples. Digital droplet PCRs employing isolated gDNA as template were prevalidated assays executed by Stem Genomics.

Immunofluorescence-based Methods

Sample preparation for intracellular staining employed a commercial buffer set according to the manufacturer's instructions (Miltenyi Biotec # 130-122-981), while using 0.5-2 M harvested cells as input and performing incubations on a sample rotator placed into a refrigerator. For surface marker staining, 0.05-0.5 M suspension cells of the immune cell lineage were merely pelleted, washed once with PBS, resuspended for staining in 50-100 µl PBS containing antibodies for 20 min, washed once more, and finally resuspended in 200-300 µl PBS. Target-specific antibodies and control IgGs are given in Table S3. Under R&D conditions, stained cells were analyzed on a MACSQuant Analyzer 16 (Miltenyi Biotec) with adjusted laser settings for each cell type. Intact single cells were pre-gated based on side vs. forward scatter as well as scatter area vs. height. Optionally, dead cells were additionally discriminated by staining with propidium iodide (1 µg/ml added to PBS for resuspension). Appropriate negative controls given in Table S3 served to set the final gates for % positive cells. In case of clearly separated positive and negative fractions in a given sample, the gate was instead set at the minimum signal in-between those populations.

Immunofluorescence microscopy was carried out according to standard procedures as described.[13] Briefly, adherent cells were fixed with 4% formaldehyde and permeabilized with 0.2% Triton X-100 during the blocking step. Primary antibodies were incubated over night at 2-8°C and Hoechst was used for DNA counterstaining. Primary and secondary antibodies used are in Table S3.

Specific Analytical Methods

For killing assays, target K562 cells (strain AAC10) were resuspended in 2.5 µM Cell Trace Violet (CTV, ThermoFisher # C34564) in PBS at 1 M/ml for 20 min at 37°C. The staining reaction was stopped by adding a 5-fold volume of hPL-based medium (see above) for 5 minutes at 37°C. The labeled cells were then resuspended in fresh hPL-based medium at 0.5 M/ml. Unlabeled iPSC-NK cells were resuspended in the same way. These two groups were mixed at 1:5, 1:1, and 5:1 ratios of NK:K562 in a final volume of 225 µl in a round-bottom 96-well plate. A 100% K562 control condition was included. The cells were briefly spun down and then incubated at 37°C. After 3.5 hr, 30 µl of 20 µM CellEvent Caspase-3/7 Green (ThermoFisher # C10423) dissolved in PBS was mixed into each well, and the cells again spun down and incubated at 37°C. 5 minutes before the end of a 4 hr incubation time, 30 µl of 10 µM SYTOX 7AAD (ThermoFisher # S10274) in PBS was mixed into each well. The plate was then analyzed by flow cytometry using a Miltenyi MACS Quant10 device. Gating strategy (established on K562 control cells): P1: all events in the forward/side scatter plot minus the CTV-negative debris. P2: Doublet exclusion. P3: CTV-positive cells. P4/5: Caspase-3/7 green on one axis, 7AAD on the other, measuring caspase single-positive cells and caspase/7AAD double-positive cells relative to the K562 control group which can be up to 5% positive.

For transepithelial electrical resistance (TEER) measurements as well as for preparing samples for electron microscopy, iPSC-RPE cells were reseeded onto transwells (Costar # 3470 ; 0.33 cm^2 surface area, coated with 0.5 µl iMatrix-511 per well; Seeding density and medium as above) ~6. The RPE

cells were then allowed to maturate under these conditions for up to 4 weeks. TEER measurements were conducted using an EVOM device (World Precision Instruments) while inserting the long electrode into the bottom reservoir and the short electrode into the upper one. Manually scraped cell layers served as baseline control. Normalized TEER values were obtained by multiplying resistance by the surface area of a given cell layer.

For scanning electron microscopy (SEM), iPSC-derived RPE were fixed in 2.5% glutaraldehyde (Sigma, Germany) in 0.1M cacodylate buffer pH 7.4 (Sciences Services, Germany) for 2 h at room temperature (RT), washed twice in 0.1M cacodylate buffer pH 7.4 and dehydrated in a graded ethanol series. The samples were critical point-dried in 100% ethanol (CPD 300, Leica, Austria) and then mounted on Leit-Tabs (Plano, Germany) glued on aluminum stubs (Plano, Germany) and sputter-coated with a 10 nm thin gold layer (ACD600, Leica, Austria). SEM images were taken using a JEOL JSM-IT200 SEM (JEOL, Japan) at 15kV with a SE detector.

For transmission electron microscopy (TEM), iPSC-derived RPE cells were fixed for 2 h at room temperature (RT) in 2.5% glutaraldehyde (Sigma, Germany) in 0.1M cacodylate buffer pH 7.4 (Sciences Services, Germany), post-fixed for 2 h at RT in 0. 1M cacodylate buffer pH 7.4, postfixed for 2 h at RT in 1% osmium tetroxide (Sciences Services, Germany) in 0.1M cacodylate buffer pH 7.4, dehydrated in a graded ethanol series and embedded in Epon 812 (Fluka, Buchs, Switzerland). Ultrathin sections (70 nm, ultramicrotome EM UC7, Leica, Wetzlar, Germany) were then stained in 1% aqueous uranyl acetate (Leica, Germany) for 30 min and in 3% lead citrate (Leica, Germany) for 20 min. TEM images were taken using a Zeiss 902 transmission electron microscope.

Statistical Methods
Quantitative data were processed in MS Excel or GraphPad Prism. Replicates were biological not technical ones throughout. Error bars in charts highlighting individual replicates indicate 95% confidence intervals of the mean values. Otherwise, error bars denote standard deviations. Statistical significance was assessed using built-in tools in Metascape or using 2-sided paired or unpaired t-tests against a given control sample as appropriate. P values of <0.05 (*) or <0.01 (**) highlight statistically significant differences.

For calculating the likelihood of identifying suitable patients for a given HLA-homozygous iPSC line, frequency data of all known haplotypes in various ethnicities were downloaded from the Be The Match Registry.[36] This comprised haplotype frequency data covering HLA class I and II combined (5 loci), HLA class I (3 loci - ABC), or HLA-C data only. The different haplotype frequency tables were ranked in descending order to then calculate cumulative frequencies in each row. For assessing a real set of HLAh iPSC lines, rows with haplotypes not covered by the iPSCs were simply deleted from a given table and cumulative frequencies recalculated. "Matchability" of a given HLA-homozygous iPSC line to a given population is unequal to the haplotype frequency because diploidy allows for 2 "matching trials". Hence, a Bernoulli process (Binomial distribution) of binary outcomes ("match" vs. "no match") with 2 trials (= alleles) was used as a model for calculating the likelihood of either matching 1 or 2 alleles ("matchability") in a random individual using a given HLAh iPSC line. Likelihood for 1 *or* 2 matched alleles = 1 - P_0 (likelihood for 0 matches: k = 0), and $P_0 = (1-p)^n$ = $(1 - \text{haplotype frequency})^{2\ (\text{number of trials/alleles})}$. Cumulative probabilities for haplotype matching using a set of - theoretical or existing - cell lines were accordingly calculated row-by-row in the ranked tables based on the previously calculated cumulative haplotype frequencies. Numerical results using this approach were in agreement with data in Xu et al.[16]

REFERENCES

11. Liedtke S, Korschgen L, Korn J et al. GMP-grade CD34(+) selection from HLA-homozygous licensed cord blood units and short-term expansion under European ATMP regulations. **Vox Sang**. 2021;116:123-135.
12. Okita K, Yamakawa T, Matsumura Y et al. An efficient nonviral method to generate integration-free human-induced pluripotent stem cells from cord blood and peripheral blood cells. **Stem Cells**. 2013;31:458-466.
13. Rao J, Pfeiffer MJ, Frank S et al. Stepwise Clearance of Repressive Roadblocks Drives Cardiac Induction in Human ESCs. **Cell Stem Cell**. 2016;18:341-353.
16. Xu H, Wang B, Ono M et al. Targeted Disruption of HLA Genes via CRISPR-Cas9 Generates iPSCs with Enhanced Immune Compatibility. **Cell Stem Cell**. 2019;24:566-578 e567.
17. D'Antonio M, Benaglio P, Jakubosky D et al. Insights into the Mutational Burden of Human Induced Pluripotent Stem Cells from an Integrative Multi-Omics Approach. **Cell Rep**. 2018;24:883-894.
19. Sondka Z, Bamford S, Cole CG et al. The COSMIC Cancer Gene Census: describing genetic dysfunction across all human cancers. **Nat Rev Cancer**. 2018;18:696-705.
20. Maruotti J, Sripathi SR, Bharti K et al. Small-molecule-directed, efficient generation of retinal pigment epithelium from human pluripotent stem cells. **Proc Natl Acad Sci U S A**. 2015;112:10950-10955.
34. Vogeley C, Nguyen T, Woeste S et al. Rapid and simple analysis of short and long sequencing reads using Duesselpore(TM). **Front Genet**. 2022;13:931996.
35. Zhou Y, Zhou B, Pache L et al. Metascape provides a biologist-oriented resource for the analysis of systems-level datasets. **Nat Commun**. 2019;10:1523.
36. Gragert L, Madbouly A, Freeman J et al. Six-locus high resolution HLA haplotype frequencies derived from mixed-resolution DNA typing for the entire US donor registry. **Hum Immunol**. 2013;74:1313-1320.

SUPPLEMENTARY TABLE LEGENDS AND FIGURES

Table S1. QC testing of starting material and iPSCs.

Table S2. RNA-seq data of undifferentiated iPSCs (line R26) and five differentiated derivatives. Data were sorted by expression ratios comparing a given cell type against all others (cutoff: Top 40 markers for each cell type). Primary data denote sequencing reads mapped to given gene.

Table S3. Antibodies used in this study.

Table S4. Nucleic acid sequences.

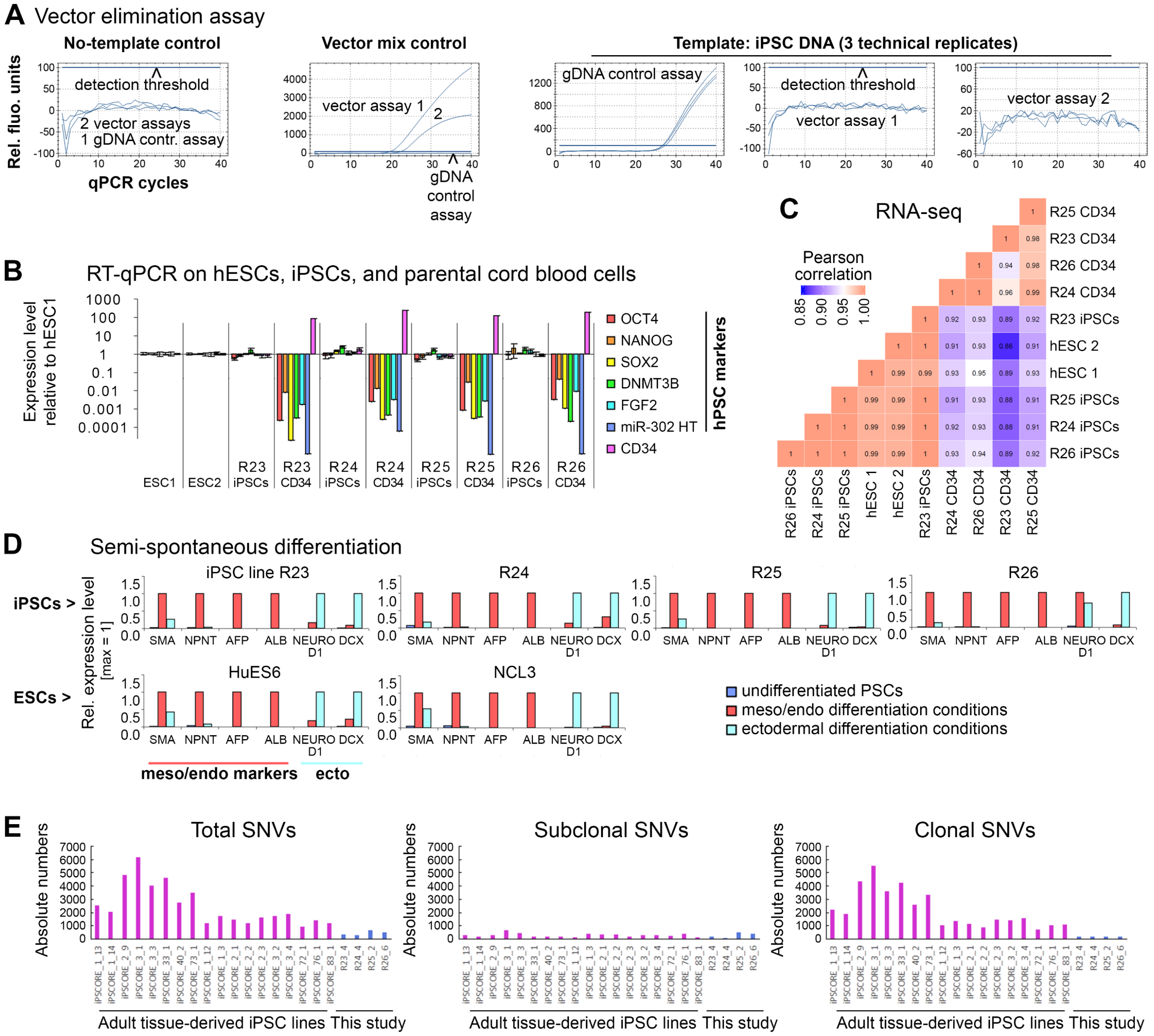

Figure S1. Additional QC testing of GMP iPSC lines. **A**, Representative qPCR plots of reprogramming vector clearance assay which consists of two vector-specific PCRs plus gDNA template control. **B**, RT-qPCR analysis of marker genes across 4 independent donors (n=3 for iPSC samples). **C**, Heat map of global correlation coefficients based on pairwise transcriptome comparisons. **D**, RT-qPCR readout of semi-spontaneous differentiation assay. Note that meso and ndodermal markers were preferentially upregulated in the mesendo-biased differentiation condition and neural markers in the more ectodermal one, as expected. **E**, Meta analysis of global mutation load in adult tissue-derived iPS cells[17] and the 4 lines of the present study. Bulk DNA of the starting material served as baseline for mutation identification. Clonal single-nucleotide variations were defined as affecting around 50% of the sequencing reads, i.e., heterozygosity in all cells.

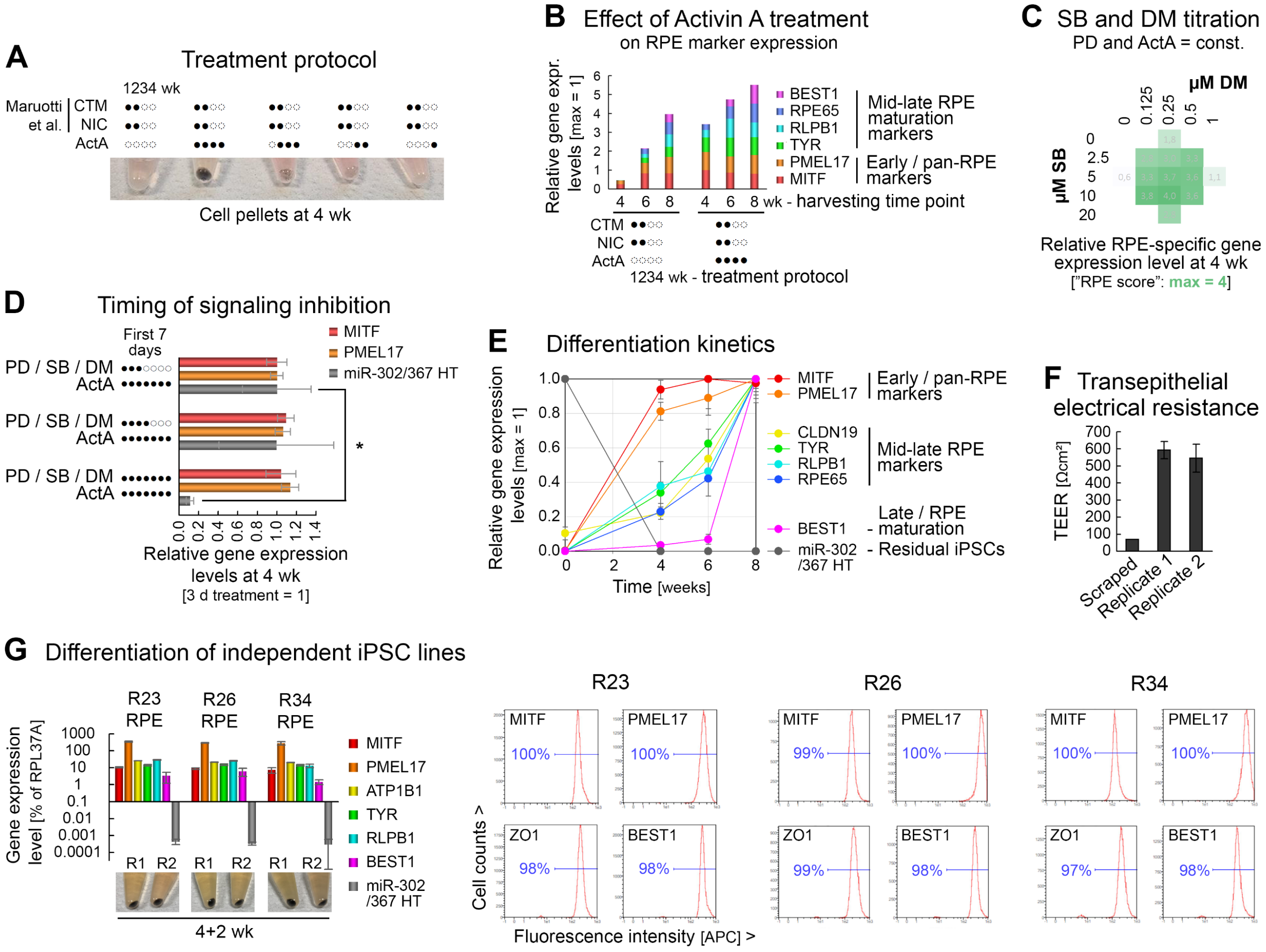

Figure S2. Directed RPE differentiation across iPSC lines. **A**, Activin A (ActA) supplementation enhances RPE induction in a published protocol. CTM: Chetomin, NIC: Nicotinamide. **B**, Quantification of marker gene expression levels in the indicated samples at different time points (exemplary experiment). **C**, Heat map of grid-type dose optimization of 2 of the induction molecules (averaged RT-qPCR data of 4 stable RPE markers at 4 wk, n=4-5 per data point). **D**, Prolonged treatment of iPSCs with the small-molecules does not enhance RPE induction but more effectively diminishes residual iPSC expression (RT-qPCR data, n=3). **E**, Differential induction of RPE genes in new differentiation protocol over time. The markers may be classified according to their induction kinetics as shown (n=2-15 per data point). **F**, Repeated TEER measurements of different preparations indicating transepithelial barrier function by iPSC-RPE layers (n=4). Data in A-F: iPSC line R26. **G**, iPS cell line independence of differentiation protocol (RT-qPCR data, n=2, and pigmentation phenotype in cell pellets). **H**, Characterization of RPE cells derived from independent iPSC lines at protein level (representative flow cytometry data).

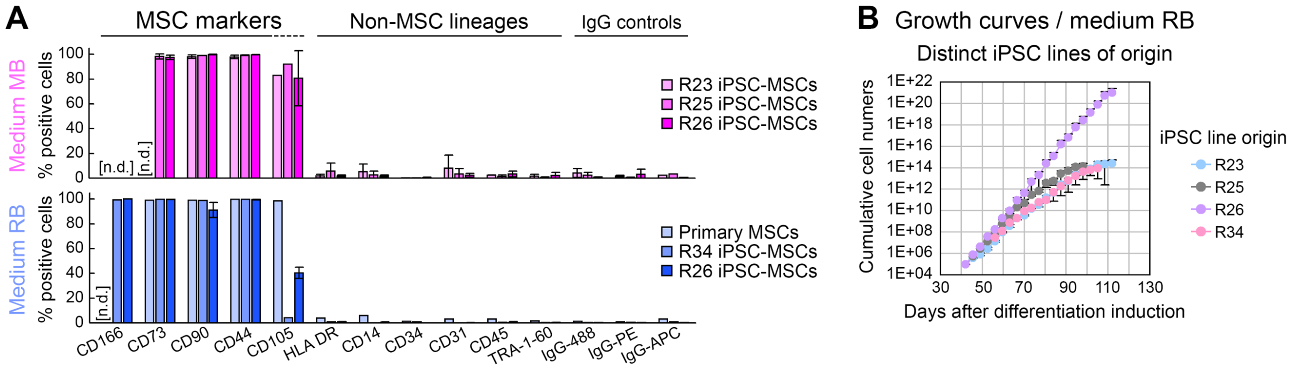

Figure S3. Donor-independent iPSC-MSC induction. **A**, Flow cytometry-based profiling of iPSC-MSCs from distinct donors and derived in different media (n=1-3). CD105 expression was somewhat more consistent in medium MB. **B**, Medium RB-based growth curves of iPSC-MSCs derived from independent iPSC lines (n=1-4).

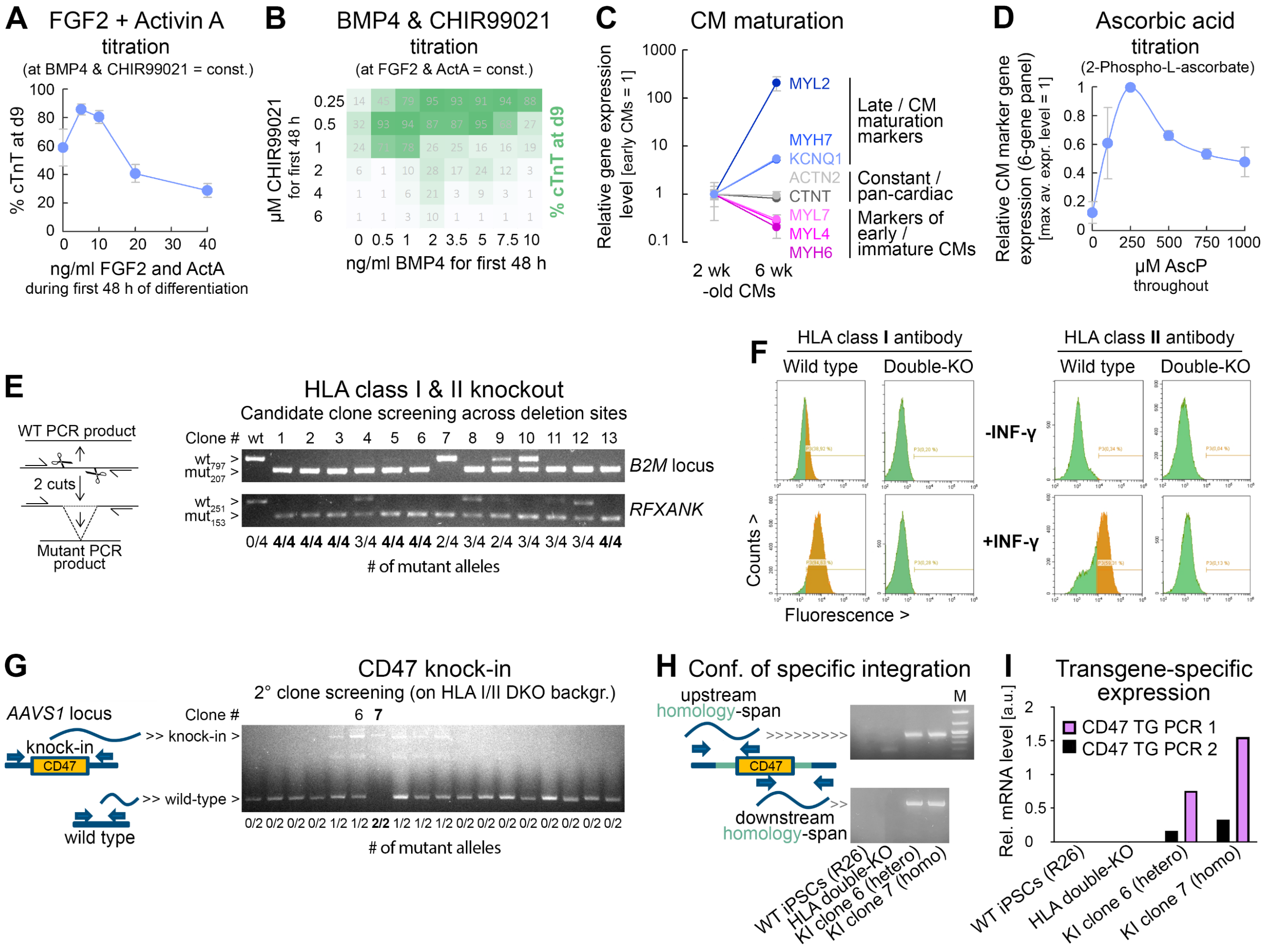

Figure S4. Supporting optimization and characterization data underlying cardiac differentiation and enhanced gene editing procedures. **A**, Titration of accessory factors in semi-optimized 3D cardiac induction protocol (n=8-14 per data point). Note the enhanced CM differentiation efficiencies with moderate FGF2 + ActA supplementation and the drop at higher doses. Titration of the two factors against each other did not reveal additional benefit. **B**, Heat map representation of grid-type titration of BMP4 and CHIR99021 concentrations for optimizing cardiac mesoderm induction in 3D (flow cytometry data, n=1-3 per data point). **C**, RT-qPCR analysis confirming expected temporal patterns of early/immature (pink), pan/stable (grey), and maturation markers of iPSC-CMs in 3D (n=2). Note the exchange of myosin light and heavy chain-encoding genes by alternative variants. **D**, 2-phospho-L-ascorbate revealed as a critical factor for cardiac differentiation from iPSCs, with striking dose-dependency (averaged RT-qPCR data, n=2). **E**, PCR-based screening strategy of HLA-controlling loci targeted with 2 gRNAs each (left) and results for randomly isolated iPSC clones (right). **F**, Flow cytometry-based analysis of HLA class I and II expression in wild-type iPSCs and progeny (line R26) as well as in cells of a selected double-knockout iPSC clone. INF-γ served to upregulate HLA levels above default levels. The cells underlying the HLA II panel were iPSC-derived endothelial cells differentiated according to Figs. 5 and S5 because undifferentiated iPSCs did not express sufficient amounts neither without nor with INF-γ. Note the complete absence of HLA expression at protein level in the double-knockout cells even after INF-γ stimulation. **G**, Illustration of targeting a *CD47* transgene to the *AAVS1* locus and PCR-based screening of isolated candidate clones after applying semi-optimized nucleofection conditions. Clones # 6 (heterozygous) and # 7 (homozygous) were selected and expanded for further characterization. **H**, Confirmation of specific transgene integration in clones 6 and 7 by conventional homology arm-spanning PCR. **I**, Transgene-specific RT-qPCR data indicating transgene dosage-dependent expression levels in the iPSC ground state, i.e. approximately 2 x transgene-specific *CD47* in the homozygous as compared to the heterozygous line.

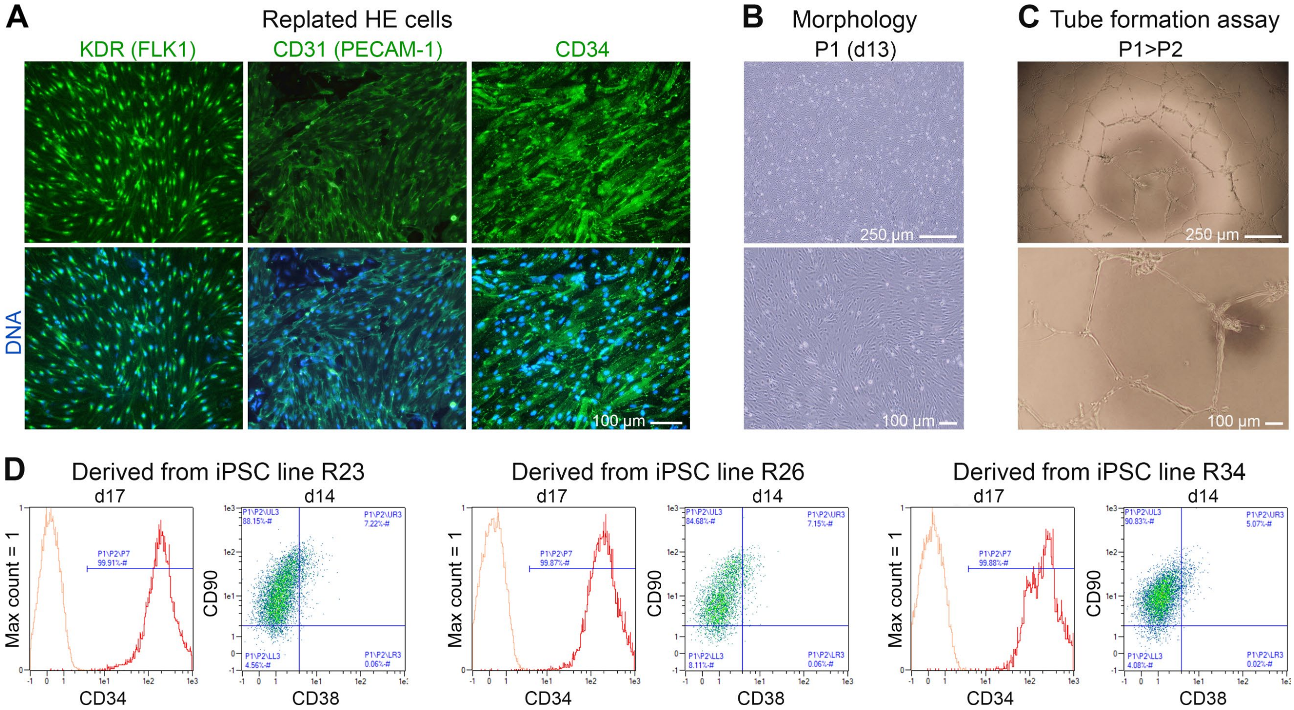

Figure S5. HE-like d7 cells mark a diverging point between endothelial and hematopoietic commitment. **A**, Immunofluorescence analysis of replated d7 cells confirming co-expression of endothelial and hematopoietic markers at this stage. **B**, HE-like cells replated onto fibronectin in the presence of VEGFA acquire an endothelial morphology. **C**, Seeding of endothelial precursor cells onto undiluted Matrigel™ promotes blood vessel-like tube formation within 24 hr. **D**, Naive CD34^{+}/CD90^{+}/CD38^{-} HSC-like expression signature of d14/17 hematopoietic precursor cells derived from independent iPSC lines under defined media conditions.

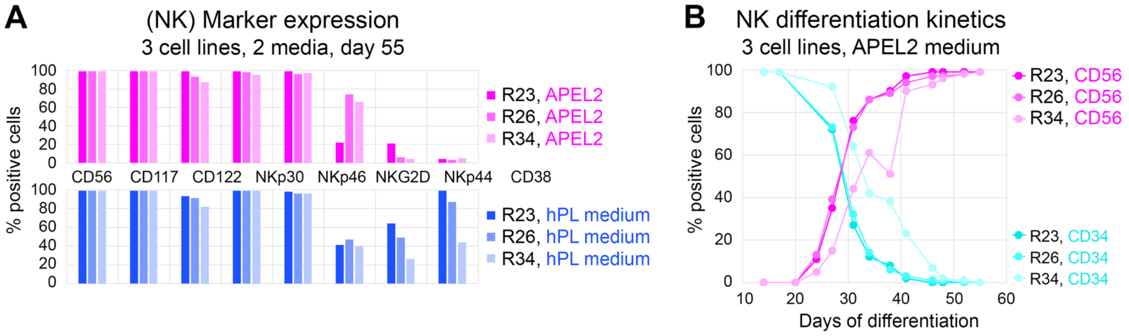

Figure S6. NK differentiation procedure is iPSC line independent. **A**, End point expression analysis by flow cytometry. Low NKp44 and CD38 levels in defined medium may indicate a more naive NK phenotype. **B**, Differentiation time course analysis in chemically defined medium using 3 independent GMP iPSC lines (flow cytometry data). Note the critical transition point from precursors to NK cells around day 28.
