# Supplementary material for: GMP-compliant iPS cell lines show widespread plasticity in a new set of differentiation workflows for cell replacement and cancer immunotherapy
Source: Stem Cells Transl Med. 2024 Jul 23;13(9):898–911. doi: 10.1093/stcltm/szae047 (PMC11386223; doi:10.1093/stcltm/szae047)
Supplement: szae047_suppl_Supplementary_Table_S1 [file szae047_suppl_supplementary_table_s1.pdf]

Specific safety testing specifications concerning starting material

Testing of mother

	Specification	***Remarks***
α-HBc-Ab	Negative	
α-HCV-Ab	Negative	
HEV-NAT	Negative	For donations after 01/2020
α-HIV I/II-Ab	Negative	
α-HTLV I/II-Ab	Negative	
HBsAG	Negative	
TPPA	Negative	
HBV-PCR	Negative	Alternatively on cord blood
HCV-PCR	Negative	Alternatively on cord blood
HIV-PCR	Negative	Alternatively on cord blood
α-CMV-IgG		For information only
α-CMV-IgM		For information only

Testing of cord blood

CMV-PCR	Negative	
HBV-PCR	Negative	Alternatively on mother
HCV-PCR	Negative	Alternatively on mother
HIV-PCR	Negative	Alternatively on mother
Parvo-B19-PCR	Negative	

In-process controls (reprogramming / iPSC clone selection)

	Specification	***Assay***	***Category***
Marker gene expression	<3-fold average deviation from hESC controls	RT-qPCR on iPSC clone for 6 hPSC markers	Identity
	>10-fold average upregulation against starting cells	RT-qPCR on iPSC clone for 6 hPSC markers	Identity
	>10-fold downregulation against starting cells	RT-qPCR on iPSC clone for starting cell marker	Identity
	>70% positive for hPSC marker	Flow cytometry before cell bank cryopreservation	Identity / purity
Reprogramming vectors	Undetectable in 2 consecutive passages	2 TaqMan assays (40 cycles)	Safety

QC specifications (batch release criteria) for iPSCs

	Specification	***Assay***	***Category***
Sterility	Negative	Acc. Ph.Eur. 2.6.27 (BD BACTEX FX)	Safety
Mycoplasma	Negative	Acc. Ph.Eur. 2.6.7 (culture method)	Safety
	Negative	Acc. Ph.Eur. 2.6.7 (indicator cell method)	Safety
Endotoxin	<1.0 EU/ml	Acc. Ph.Eur. 2.6.14 (method D)	Safety
Viral contaminants	Negative	Transmission electron microscopy (TEM assay)	Safety
	Negative	Product-Enhanced Reverse-Transkriptase-Assay (PERT)	Safety
	No indication of viral replication	Deep RNA-sequencing for active viruses	Safety

Morphology	Epithelial epiblast-like, colony-forming	Phase contrast microscopy	Identity
Marker gene expression	>70% positive for 2 hPSC markers	Flow cytometry	Identity / purity
	Staining overall specific and positive for 5 hPSC markers	Immunocytochemistry	Identity
	<3-fold average deviation from hESC controls	RT-qPCR for 6 hPSC markers	Identity
Genetic identity	Identical profiles	STR analysis of iPSCs and parental CB cells	Identity
Genomic integrity	46,XX or 46,XY genotype, no aneuploidies	Cytogenetic analysis (G banding)	Integrity
	No gain of functional mutations in defined cancer gene panel	Whole-genome sequencing (iPSCs against CB)	Integrity / safety
	No gain of numerical aberrations >5 Mbp	Whole-genome sequencing (iPSCs against CB)	Integrity
iPSC recovery	>100,000 attached live cells 24h post thaw	Cell counting / Trypan Blue exclusion	Integrity
Reprogramming vectors	Undetectable	2 TaqMan assays (40 cycles)	Impurity / safety
Pluripotency	Staining specific and positive for 1 marker per germ layer	Immunocytochemistry on differentiated samples	Potency
	>5-fold upregulation vs. iPSCs for 6 marker genes	RT-qPCR on 2 marker genes per germ layer	Potency

Optional accessory testing for viral contaminants on selected EU iPSC lines according to 21 CFR 1271 requirements

	Specification	***Assay***
In vivo testing	Negative	In vivo detection of adventitious viruses (ICH/EU)
In vitro testing	Negative	In vitro detection of adventitious viruses (ICH/EU)
CMV	Negative	PCR
HIV 1&2	Negative	PCR
HTLV 1&2	Negative	PCR
HHV-6	Negative	PCR
HHV-7	Negative	PCR
HHV-8	Negative	PCR
JC Virus	Negative	PCR
BK Virus	Negative	PCR
EBV	Negative	PCR
EV (Enterovirus)	Negative	PCR
Human Parvovirus B19	Negative	PCR
HAV	Negative	PCR
HBV	Negative	PCR
HPV-16	Negative	PCR
HPV-18	Negative	PCR
HCV	Negative	PCR
WNV	Negative	PCR
ZIKV	Negative	PCR

Legend

	Definition
Ab	Detectable antibodies
BK	BK-Polyomavirus
CB	Cord blood
CMV	Cytomegalovirus
EBV	Epstein-Barr virus
HB/HC/HE	Hepatitis B/C/E virus

HBc	Hepatitis B core
HBsAG	Hepatitis B surface antigen
HIV	Human immunodeficiency viruses
HPV	Human Papillomavirus
HTLV	Human T-cell leukemia-lymphoma virus
JC	Human Polyomavirus 2
NAT	Nucleic acid test
STR	Short tandem repeat
TPPA	Treponema palladium
WNV	West Nile virus
ZIKV	Zika virus
