# Supplementary material for: GMP-compliant iPS cell lines show widespread plasticity in a new set of differentiation workflows for cell replacement and cancer immunotherapy
Source: Stem Cells Transl Med. 2024 Jul 23;13(9):898–911. doi: 10.1093/stcltm/szae047 (PMC11386223; doi:10.1093/stcltm/szae047)
Supplement: szae047_suppl_Supplementary_Table_S2 [file szae047_suppl_supplementary_table_s2.pdf]

Gene ID	Chr	WikiGene description	iPSCs # reads per mio	RPE # reads per mio	MSCs # reads per mio	CMs # reads per mio	HE # reads per mio	NK # reads per mio	Gene symbol	Ratio RPE vs others	Ratio MSCs vs others	Ratio CMs vs others	Ratio HE vs others	Ratio NK vs others
ENSG00000118271	18	transthyretin	4	6497	1	3	1	1	TTR	3248.5	0.0	0.0	0.0	0.0
ENSG00000107165	9	tyrosinase related protein 1	1	2535	1	1	1	1	TYRP1	2535.0	0.0	0.0	0.0	0.0
ENSG00000080166	13	dopachrome tautomerase	2	2245	1	1	1	1	DCT	1870.8	0.0	0.0	0.0	0.0
ENSG00000134160	15	transient receptor potential cation channel subfamily M member 1	1	1188	1	1	1	1	TRPM1	1188.0	0.0	0.0	0.0	0.0
ENSG00000253117	8	otoconin 90	1	737	1	1	1	1	OC90	737.0	0.0	0.0	0.0	0.0
ENSG00000107317	9	prostaglandin D2 synthase	2	2186	12	1	1	1	PTGDS	642.9	0.0	0.0	0.0	0.0
ENSG00000049540	7	elastin	7	2869	8	1	6	1	ELN	623.7	0.0	0.0	0.0	0.0
ENSG00000086696	16	hydroxysteroid 17-beta dehydrogenase 2	1	947	3	1	1	2	HSD17B2	591.9	0.0	0.0	0.0	0.0
ENSG00000235448	9	0	1	327	1	1	1	1	LURAP1L-AS1	327.0	0.0	0.0	0.0	0.0
ENSG00000164175	5	solute carrier family 45 member 2	1	427	3	1	1	1	SLC45A2	305.0	0.0	0.0	0.0	0.0
ENSG00000261235	16	0	3	857	1	1	3	7	HSD17B2-AS1	285.7	0.0	0.0	0.0	0.0
ENSG00000232044	2	0	1	273	1	1	1	1	SILC1	273.0	0.0	0.0	0.0	0.0
ENSG00000185664	12	premelanosome protein	111	9513	5	24	38	7	PMEL	257.1	0.0	0.0	0.0	0.0
ENSG00000077498	11	tyrosinase	1	241	1	1	1	1	TYR	241.0	0.0	0.0	0.0	0.0
ENSG00000083067	9	transient receptor potential cation channel subfamily M member 3	12	717	1	2	1	1	TRPM3	210.9	0.0	0.0	0.0	0.0
ENSG00000243566	7	uroplakin 3B	1	174	1	1	1	1	UPK3B	174.0	0.0	0.0	0.0	0.0
ENSG00000069696	11	dopamine receptor D4	2	313	5	1	1	1	DRD4	156.5	0.1	0.0	0.0	0.0
ENSG00000140522	15	retinaldehyde binding protein 1	1	155	1	1	1	1	RLBP1	155.0	0.0	0.0	0.0	0.0
ENSG00000136235	7	glycoprotein nmb	2	1165	28	1	1	7	GPNMB	149.4	0.1	0.0	0.0	0.0
ENSG00000163817	3	solute carrier family 6 member 20	2	171	1	1	1	1	SLC6A20	142.5	0.0	0.0	0.0	0.0
ENSG00000007372	11	paired box 6	1	138	1	1	1	1	PAX6	138.0	0.0	0.0	0.0	0.0
ENSG00000188505	19	NCCRP1, F-box associated domain containing	1	134	1	1	1	1	NCCRP1	134.0	0.0	0.0	0.0	0.0
ENSG00000121898	10	carboxypeptidase X, M14 family member 2	8	317	1	1	1	1	CPXM2	132.1	0.0	0.0	0.0	0.0
ENSG00000081277	1	plakophilin 1	5	232	1	1	1	1	PKP1	128.9	0.0	0.0	0.0	0.0
ENSG00000253309	13	serpin family E member 3	2	228	4	1	1	1	SERPINE3	126.7	0.1	0.0	0.0	0.0
ENSG00000198597	19	zinc finger protein 536	1	122	1	1	1	1	ZNF536	122.0	0.0	0.0	0.0	0.0
ENSG00000174808	4	betacellulin	1	121	1	1	1	1	BTC	121.0	0.0	0.0	0.0	0.0
ENSG00000237988	6	olfactory receptor family 2 subfamily I member 1 pseudogene	1	119	1	1	1	1	OR2I1P	119.0	0.0	0.0	0.0	0.0
ENSG00000177459	8	glutamate rich 5	1	111	1	1	1	1	ERICH5	111.0	0.0	0.0	0.0	0.0
ENSG00000189056	7	reelin	1	821	1	33	1	1	RELN	110.9	0.0	0.2	0.0	0.0
ENSG00000164112	4	small integral membrane protein 43	2	121	1	1	1	1	SMIM43	100.8	0.0	0.0	0.0	0.0
ENSG00000162989	2	potassium inwardly rectifying channel subfamily J member 3	1	88	1	1	1	1	KCNJ3	88.0	0.1	0.1	0.1	0.1
ENSG00000179292	11	transmembrane protein 151A	4	138	1	1	1	1	TMEM151A	86.3	0.0	0.0	0.0	0.0
ENSG00000171243	7	sclerostin domain containing 1	1	83	1	1	1	1	SOSTDC1	83.0	0.1	0.1	0.1	0.1
ENSG00000182747	6	solute carrier family 35 member D3	1	114	1	1	3	1	SLC35D3	81.4	0.0	0.0	0.1	0.0
ENSG00000259583	15	ALDH1A3 antisense RNA 1	1	85	1	1	1	2	ALDH1A3-AS1	70.8	0.1	0.1	0.1	0.1
ENSG00000100156	22	solute carrier family 16 member 8	1	68	1	1	1	1	SLC16A8	68.0	0.1	0.1	0.1	0.1
ENSG00000248550	14	0	1	67	1	1	1	1	OTX2-AS1	67.0	0.1	0.1	0.1	0.1
ENSG00000120057	10	secreted frizzled related protein 5	1	66	1	1	1	1	SFRP5	66.0	0.1	0.1	0.1	0.1
ENSG00000080644	15	cholinergic receptor nicotinic alpha 3 subunit	18	435	1	1	12	1	CHRNA3	65.9	0.0	0.0	0.1	0.0
ENSG00000108821	17	collagen type I alpha 1 chain	287	26	47194	78	96	37	COL1A1	0.0	450.3	0.0	0.0	0.0
ENSG00000133110	13	periostin	1	3	2416	1	21	1	POSTN	0.0	447.4	0.0	0.0	0.0

ENSG00000111799	6	collagen type XII alpha 1 chain	13	3	1792	1	3	1	COL12A1	0.0	426.7	0.0	0.0	0.0
ENSG00000166741	11	nicotinamide N-methyltransferase	1	1	1182	1	3	9	NNMT	0.0	394.0	0.0	0.0	0.0
ENSG00000163017	2	actin gamma 2, smooth muscle	14	1	1799	1	1	6	ACTG2	0.0	391.1	0.0	0.0	0.0
ENSG00000188624	19	IGF like family member 3	1	1	316	1	1	1	IGFL3	0.0	316.0	0.0	0.0	0.0
ENSG00000164692	7	collagen type I alpha 2 chain	132	62	38467	182	343	31	COL1A2	0.0	256.4	0.0	0.0	0.0
ENSG00000078098	2	fibroblast activation protein alpha	1	2	263	1	1	1	FAP	0.0	219.2	0.0	0.0	0.0
ENSG00000041982	9	tenascin C	11	1	721	4	1	1	TNC	0.0	200.3	0.0	0.0	0.0
ENSG00000198542	13	integrin subunit beta like 1	1	1	181	1	1	1	ITGBL1	0.0	181.0	0.0	0.0	0.0
ENSG00000186340	6	thrombospondin 2	27	2	1177	1	1	2	THBS2	0.0	178.3	0.0	0.0	0.0
ENSG00000101825	X	matrix remodeling associated 5	1	4	273	1	1	1	MXRA5	0.1	170.6	0.0	0.0	0.0
ENSG00000226445	6	0	25	4	1131	1	1	3	THBS2-AS1	0.0	166.3	0.0	0.0	0.0
ENSG00000120708	5	transforming growth factor beta induced	13	68	3416	6	12	6	TGFBI	0.1	162.7	0.0	0.0	0.0
ENSG00000115414	2	fibronectin 1	25	8	5198	25	81	21	FN1	0.0	162.4	0.0	0.1	0.0
ENSG00000102466	13	fibroblast growth factor 14	1	2	193	1	1	1	FGF14	0.1	160.8	0.0	0.0	0.0
ENSG00000197614	12	microfibril associated protein 5	1	1	158	1	1	1	MFAP5	0.0	158.0	0.0	0.0	0.0
ENSG00000139329	12	lumican	1	3	1559	11	29	7	LUM	0.0	152.8	0.0	0.1	0.0
ENSG00000116132	1	paired related homeobox 1	1	13	621	1	6	2	PRRX1	0.1	135.0	0.0	0.0	0.0
ENSG00000163359	2	collagen type VI alpha 3 chain	2	1	726	2	23	1	COL6A3	0.0	125.2	0.0	0.2	0.0
ENSG00000231290	20	APCDD1L divergent transcript	1	1	115	1	1	1	APCDD1L-DT	0.0	115.0	0.0	0.0	0.0
ENSG00000137801	15	thrombospondin 1	16	1	591	1	5	3	THBS1	0.0	113.7	0.0	0.0	0.0
ENSG00000082497	1	SERTA domain containing 4	1	1	113	1	1	1	SERTAD4	0.0	113.0	0.0	0.0	0.0
ENSG00000164932	8	collagen triple helix repeat containing 1	9	7	892	17	7	1	CTHRC1	0.0	108.8	0.1	0.0	0.0
ENSG00000082196	5	C1q and TNF related 3	1	5	433	2	8	4	C1QTNF3	0.1	108.3	0.0	0.1	0.0
ENSG00000183160	12	transmembrane protein 119	1	1	263	1	9	1	TMEM119	0.0	101.2	0.0	0.2	0.0
ENSG00000168542	2	collagen type III alpha 1 chain	3	29	8134	26	338	22	COL3A1	0.0	97.3	0.0	0.2	0.0
ENSG00000079931	6	monooxygenase DBH like 1	4	1	155	1	1	1	MOXD1	0.0	96.9	0.0	0.0	0.0
ENSG00000138316	10	ADAM metallopeptidase with thrombospondin type 1 motif 14	4	1	211	1	2	3	ADAMTS14	0.0	95.9	0.0	0.0	0.1
ENSG00000130635	9	collagen type V alpha 1 chain	6	13	1331	22	29	2	COL5A1	0.0	92.4	0.1	0.1	0.0
ENSG00000129009	15	immunoglobulin superfamily containing leucine rich repeat	1	7	386	1	11	1	ISLR	0.1	91.9	0.0	0.1	0.0
ENSG00000128422	17	keratin 17	1	3	125	1	1	1	KRT17	0.1	89.3	0.0	0.0	0.0
ENSG00000255690	7	TLR4 interactor with leucine rich repeats	1	1	98	1	2	1	TRIL	0.0	81.7	0.0	0.1	0.0
ENSG00000173077	9	0	1	1	81	1	1	1	DELEC1	0.1	81.0	0.1	0.1	0.1
ENSG00000182492	X	biglycan	4	15	2744	1	154	4	BGN	0.0	77.1	0.0	0.3	0.0
ENSG00000126778	14	SIX homeobox 1	1	1	75	1	1	1	SIX1	0.1	75.0	0.1	0.1	0.1
ENSG00000011465	12	decorin	1	4	116	1	1	1	DCN	0.2	72.5	0.0	0.0	0.0
ENSG00000128710	2	homeobox D10	1	1	71	1	1	1	HOXD10	0.1	71.0	0.1	0.1	0.1
ENSG00000128713	2	homeobox D11	1	1	71	1	1	1	HOXD11	0.1	71.0	0.1	0.1	0.1
ENSG00000137809	15	integrin subunit alpha 11	5	2	298	7	3	4	ITGA11	0.0	71.0	0.1	0.0	0.1
ENSG00000197616	14	myosin heavy chain 6	1	1	1	7819	3	1	MYH6	0.0	0.0	5585.0	0.0	0.0
ENSG00000106631	7	myosin light chain 7	8	2	1	6132	2	1	MYL7	0.0	0.0	2190.0	0.0	0.0
ENSG00000198336	17	myosin light chain 4	1	4	13	4598	4	1	MYL4	0.0	0.0	999.6	0.0	0.0
ENSG00000159173	1	troponin I1, slow skeletal type	2	9	1	2492	1	1	TNNI1	0.0	0.0	890.0	0.0	0.0
ENSG00000092054	14	myosin heavy chain 7	1	1	1	741	1	1	MYH7	0.0	0.0	741.0	0.0	0.0
ENSG00000160808	3	myosin light chain 3	2	1	1	2225	11	1	MYL3	0.0	0.0	695.3	0.0	0.0
ENSG00000205678	4	trans-2,3-enoyl-CoA reductase like	1	1	1	526	1	1	TECRL	0.0	0.0	526.0	0.0	0.0
ENSG00000118194	1	troponin T2, cardiac type	11	8	5	2476	2	1	TNNT2	0.0	0.0	458.5	0.0	0.0
ENSG00000140795	16	myosin light chain kinase 3	4	2	1	663	2	1	MYLK3	0.0	0.0	331.5	0.0	0.0
ENSG00000134571	11	myosin binding protein C3	1	1	1	281	1	1	MYBPC3	0.0	0.0	281.0	0.0	0.0
ENSG00000198125	22	myoglobin	1	2	1	497	1	4	MB	0.0	0.0	276.1	0.0	0.0
ENSG00000173641	1	heat shock protein family B (small) member 7	1	4	1	424	1	1	HSPB7	0.0	0.0	265.0	0.0	0.0
ENSG00000141161	17	unc-45 myosin chaperone B	1	1	1	362	1	3	UNC45B	0.0	0.0	258.6	0.0	0.0

ENSG00000122367	10	LIM domain binding 3	1	1	1	256	1	1	LDB3	0.0	0.0	256.0	0.0	0.0
ENSG00000139914	14	fat storage inducing transmembrane protein 1	1	4	2	443	1	3	FITM1	0.0	0.0	201.4	0.0	0.0
ENSG00000250007	15	0	11	9	3	1116	4	1	GJD2-DT	0.0	0.0	199.3	0.0	0.0
ENSG00000159251	15	actin alpha cardiac muscle 1	13	9	2	1186	5	1	ACTC1	0.0	0.0	197.7	0.0	0.0
ENSG00000115593	2	SET and MYND domain containing 1	1	1	1	177	1	1	SMYD1	0.0	0.0	177.0	0.0	0.0
ENSG00000129170	11	cysteine and glycine rich protein 3	1	1	1	176	1	1	CSRP3	0.0	0.0	176.0	0.0	0.0
ENSG00000113430	5	iroquois homeobox 4	1	1	1	151	1	1	IRX4	0.0	0.0	151.0	0.0	0.0
ENSG00000183072	5	NK2 homeobox 5	1	1	1	143	1	1	NKX2-5	0.0	0.0	143.0	0.0	0.0
ENSG00000078114	10	nebulette	31	5	1	1231	11	1	NEBL	0.0	0.0	125.6	0.0	0.0
ENSG00000104879	19	creatine kinase, M-type	1	1	1	143	1	2	CKM	0.0	0.0	119.2	0.0	0.1
ENSG00000130528	19	histidine rich calcium binding protein	1	1	1	112	1	1	HRC	0.0	0.0	112.0	0.0	0.0
ENSG00000141052	17	myocardin	4	7	1	311	1	1	MYOCD	0.1	0.0	111.1	0.0	0.0
ENSG00000091482	X	small muscle protein X-linked	1	5	1	197	1	1	SMPX	0.1	0.0	109.4	0.0	0.0
ENSG00000143171	1	retinoid X receptor gamma	1	3	1	153	1	1	RXRG	0.1	0.0	109.3	0.0	0.0
ENSG00000077522	1	actinin alpha 2	3	1	1	147	1	1	ACTN2	0.0	0.0	105.0	0.0	0.0
ENSG00000172399	4	myozenin 2	1	1	1	98	1	1	MYOZ2	0.0	0.0	98.0	0.0	0.0
ENSG00000114854	3	troponin C1, slow skeletal and cardiac type	6	81	2	1792	8	1	TNNC1	0.2	0.0	91.4	0.0	0.0
ENSG00000169436	8	collagen type XXII alpha 1 chain	11	16	3	864	19	1	COL22A1	0.1	0.0	86.4	0.1	0.0
ENSG00000101605	18	myomesin 1	1	1	8	444	1	15	MYOM1	0.0	0.1	85.4	0.0	0.2
ENSG00000156885	16	cytochrome c oxidase subunit 6A2	1	1	1	164	1	6	COX6A2	0.0	0.0	82.0	0.0	0.2
ENSG00000147573	8	tripartite motif containing 55	1	1	1	81	1	1	TRIM55	0.1	0.1	81.0	0.1	0.1
ENSG00000185739	16	sarcalumenin	1	1	1	111	3	1	SRL	0.0	0.0	79.3	0.1	0.0
ENSG00000170893	3	thyrotropin releasing hormone	1	6	1	281	9	1	TRH	0.1	0.0	78.1	0.2	0.0
ENSG00000120457	11	potassium inwardly rectifying channel subfamily J member 5	1	2	1	92	1	1	KCNJ5	0.1	0.1	76.7	0.1	0.1
ENSG00000136574	8	GATA binding protein 4	1	1	1	75	1	1	GATA4	0.1	0.1	75.0	0.1	0.1
ENSG00000112175	6	bone morphogenetic protein 5	1	1	1	256	14	1	BMP5	0.0	0.0	71.1	0.3	0.0
ENSG00000166090	14	interleukin 25	1	1	1	71	1	1	IL25	0.1	0.1	71.0	0.1	0.1
ENSG00000143839	1	renin	1	1	1	1	1649	1	REN	0.0	0.0	0.0	1649.0	0.0
ENSG00000187513	1	gap junction protein alpha 4	1	1	1	3	517	1	GJA4	0.0	0.0	0.0	369.3	0.0
ENSG00000130300	19	plasmalemma vesicle associated protein	1	1	1	2	342	1	PLVAP	0.0	0.0	0.0	285.0	0.0
ENSG00000128917	15	delta like canonical Notch ligand 4	1	1	1	2	311	1	DLL4	0.0	0.0	0.0	259.2	0.0
ENSG00000149564	11	endothelial cell adhesion molecule	1	1	1	4	412	1	ESAM	0.0	0.0	0.0	257.5	0.0
ENSG00000161940	17	BCL6B transcription repressor	3	1	1	6	585	2	BCL6B	0.0	0.0	0.1	225.0	0.0
ENSG00000102924	16	cerebellin 1 precursor	3	1	1	1	287	1	CBLN1	0.0	0.0	0.0	205.0	0.0
ENSG00000100985	20	matrix metallopeptidase 9	3	1	1	1	272	1	MMP9	0.0	0.0	0.0	194.3	0.0
ENSG00000164326	5	CART prepropeptide	1	1	1	1	169	1	CARTPT	0.0	0.0	0.0	169.0	0.0
ENSG00000204103	20	MAF bZIP transcription factor B	1	2	1	1	168	1	MAFB	0.1	0.0	0.0	140.0	0.0
ENSG00000185559	14	delta like non-canonical Notch ligand 1	3	4	1	1	277	1	DLK1	0.1	0.0	0.0	138.5	0.0
ENSG00000152910	16	contactin associated protein family member 4	1	1	1	1	121	1	CNTNAP4	0.0	0.0	0.0	121.0	0.0
ENSG00000280623	22	endogenous retrovirus group K member 5 Gag polyprotein-like	1	1	1	1	118	1	PCAT14	0.0	0.0	0.0	118.0	0.0
ENSG00000174059	1	CD34 molecule	1	1	1	3	155	1	CD34	0.0	0.0	0.1	110.7	0.0
ENSG00000169291	1	Src homology 2 domain containing E	1	1	1	1	123	2	SHE	0.0	0.0	0.0	102.5	0.1
ENSG00000167244	11	insulin like growth factor 2	1	11	22	41	1594	4	IGF2	0.0	0.1	0.1	100.9	0.0
ENSG00000010319	3	semaphorin 3G	5	1	1	1	172	1	SEMA3G	0.0	0.0	0.0	95.6	0.0
ENSG00000179776	16	cadherin 5	1	1	1	4	149	1	CDH5	0.0	0.0	0.1	93.1	0.0
ENSG00000178726	20	thrombomodulin	1	1	1	3	141	2	THBD	0.0	0.0	0.1	88.1	0.1
ENSG00000175874	2	cellular repressor of E1A stimulated genes 2	1	1	1	1	88	1	CREG2	0.1	0.1	0.1	88.0	0.1
ENSG00000157554	21	ETS transcription factor ERG	2	1	1	5	173	1	ERG	0.0	0.0	0.1	86.5	0.0
ENSG00000139567	12	activin A receptor like type 1	1	1	4	1	134	1	ACVRL1	0.0	0.1	0.0	83.8	0.0
ENSG00000036565	8	solute carrier family 18 member A1	1	1	1	1	77	1	SLC18A1	0.1	0.1	0.1	77.0	0.1
ENSG00000136630	1	H2.0 like homeobox	1	1	7	1	158	1	HLX	0.0	0.2	0.0	71.8	0.0

ENSG00000125810	20	CD93 molecule	1	1	1	2	256	13	CD93	0.0	0.0	0.0	71.1	0.2
ENSG00000155792	8	DEP domain containing MTOR interacting protein	6	1	4	4	241	2	DEPTOR	0.0	0.1	0.1	70.9	0.0
ENSG00000113389	5	natriuretic peptide receptor 3	1	1	2	1	83	1	NPR3	0.1	0.1	0.1	69.2	0.1
ENSG00000164683	8	hes related family bHLH transcription factor with YRPW motif 1	1	1	1	12	219	1	HEY1	0.0	0.0	0.3	68.4	0.0
ENSG00000164736	8	SRY-box transcription factor 17	1	1	1	1	67	1	SOX17	0.1	0.1	0.1	67.0	0.1
ENSG00000170689	17	homeobox B9	1	1	27	1	411	1	HOXB9	0.0	0.3	0.0	66.3	0.0
ENSG00000129965	11	INS-IGF2 readthrough	1	1	7	9	243	1	INS-IGF2	0.0	0.1	0.2	63.9	0.0
ENSG00000037280	5	fms related receptor tyrosine kinase 4	4	1	1	2	113	1	FLT4	0.0	0.0	0.1	62.8	0.0
ENSG00000147113	X	divergent protein kinase domain 2B	1	1	1	1	62	1	DIPK2B	0.1	0.1	0.1	62.0	0.1
ENSG00000142748	1	ficolin 3	1	1	1	4	96	1	FCN3	0.0	0.0	0.2	60.0	0.0
ENSG00000171056	8	SRY-box transcription factor 7	3	1	1	16	253	1	SOX7	0.0	0.0	0.3	57.5	0.0
ENSG00000134830	19	complement C5a receptor 2	2	1	1	1	66	1	C5AR2	0.1	0.1	0.1	55.0	0.1
ENSG00000173269	10	multimerin 2	2	1	1	3	85	1	MMRN2	0.1	0.1	0.2	53.1	0.1
ENSG00000120279	6	MYC target 1	1	1	2	1	63	1	MYCT1	0.1	0.1	0.1	52.5	0.1
ENSG00000257551	1	HLX antisense RNA 1	1	1	2	1	63	1	HLX-AS1	0.1	0.1	0.1	52.5	0.1
ENSG00000141314	17	rhomboid like 3	1	1	1	1	52	1	RHBDL3	0.1	0.1	0.1	52.0	0.1
ENSG00000115523	2	granulysin	3	2	2	1	1	7662	GNLY	0.0	0.0	0.0	0.0	4256.7
ENSG00000105374	19	natural killer cell granule protein 7	1	3	1	1	1	4911	NKG7	0.0	0.0	0.0	0.0	3507.9
ENSG00000271503	17	C-C motif chemokine ligand 5	1	1	1	1	1	2625	CCL5	0.0	0.0	0.0	0.0	2625.0
ENSG00000111796	12	killer cell lectin like receptor B1	1	1	1	2	1	2186	KLRB1	0.0	0.0	0.0	0.0	1821.7
ENSG00000011600	19	transmembrane immune signaling adaptor TYROBP	1	1	1	1	1	1157	TYROBP	0.0	0.0	0.0	0.0	1157.0
ENSG00000145649	5	granzyme A	1	1	2	1	2	1377	GZMA	0.0	0.0	0.0	0.0	983.6
ENSG00000140678	16	integrin subunit alpha X	1	1	1	1	1	918	ITGAX	0.0	0.0	0.0	0.0	918.0
ENSG00000277089	17	0	1	1	1	1	1	914	CCL3-AS1	0.0	0.0	0.0	0.0	914.0
ENSG00000277632	17	C-C motif chemokine ligand 3	1	1	1	1	1	911	CCL3	0.0	0.0	0.0	0.0	911.0
ENSG00000167286	11	CD3 delta subunit of T-cell receptor complex	1	1	1	1	2	897	CD3D	0.0	0.0	0.0	0.0	747.5
ENSG00000180644	10	perforin 1	1	1	1	1	2	816	PRF1	0.0	0.0	0.0	0.0	680.0
ENSG00000227507	6	lymphotoxin beta	1	6	1	1	4	1738	LTB	0.0	0.0	0.0	0.0	668.5
ENSG00000172543	11	cathepsin W	2	3	5	7	4	2713	CTSW	0.0	0.0	0.0	0.0	646.0
ENSG00000139626	12	integrin subunit beta 7	4	2	3	1	2	1498	ITGB7	0.0	0.0	0.0	0.0	624.2
ENSG00000204475	6	natural cytotoxicity triggering receptor 3	1	1	1	1	5	978	NCR3	0.0	0.0	0.0	0.0	543.3
ENSG00000147168	X	interleukin 2 receptor subunit gamma	1	1	1	1	1	519	IL2RG	0.0	0.0	0.0	0.0	519.0
ENSG00000110934	12	bridging integrator 2	1	1	1	1	1	494	BIN2	0.0	0.0	0.0	0.0	494.0
ENSG00000205810	12	killer cell lectin like receptor C3	1	1	1	1	1	486	KLRC3	0.0	0.0	0.0	0.0	486.0
ENSG00000128340	22	Rac family small GTPase 2	1	1	1	2	4	837	RAC2	0.0	0.0	0.0	0.0	465.0
ENSG00000000938	1	FGR proto-oncogene, Src family tyrosine kinase	1	1	1	1	1	439	FGR	0.0	0.0	0.0	0.0	439.0
ENSG00000020633	1	RUNX family transcription factor 3	1	1	1	1	1	416	RUNX3	0.0	0.0	0.0	0.0	416.0
ENSG00000198851	11	CD3 epsilon subunit of T-cell receptor complex	1	1	1	1	1	399	CD3E	0.0	0.0	0.0	0.0	399.0
ENSG00000158869	1	Fc epsilon receptor Ig	2	2	2	1	3	788	FCER1G	0.0	0.0	0.0	0.0	394.0
ENSG00000204482	6	leukocyte specific transcript 1	1	1	1	1	8	937	LST1	0.0	0.0	0.0	0.0	390.4
ENSG00000132965	13	arachidonate 5-lipoxygenase activating protein	1	2	5	1	1	771	ALOX5AP	0.0	0.0	0.0	0.0	385.5
ENSG00000100385	22	interleukin 2 receptor subunit beta	2	1	1	1	6	815	IL2RB	0.0	0.0	0.0	0.0	370.5
ENSG00000143851	1	protein tyrosine phosphatase non-receptor type 7	1	1	1	1	5	657	PTPN7	0.0	0.0	0.0	0.0	365.0
ENSG00000186517	1	Rho GTPase activating protein 30	1	1	1	1	1	355	ARHGAP30	0.0	0.0	0.0	0.0	355.0
ENSG00000172236	16	tryptase alpha/beta 1	1	1	1	1	1	327	TPSAB1	0.0	0.0	0.0	0.0	327.0
ENSG00000117091	1	CD48 molecule	1	1	1	1	1	326	CD48	0.0	0.0	0.0	0.0	326.0
ENSG00000100453	14	granzyme B	1	1	1	1	1	311	GZMB	0.0	0.0	0.0	0.0	311.0
ENSG00000197253	16	tryptase beta 2	1	1	1	1	1	311	TPSB2	0.0	0.0	0.0	0.0	311.0
ENSG00000105492	19	sialic acid binding Ig like lectin 6	1	1	1	1	1	287	SIGLEC6	0.0	0.0	0.0	0.0	287.0
ENSG00000205045	17	schlafen family member 12 like	1	1	1	1	1	285	SLFN12L	0.0	0.0	0.0	0.0	285.0
ENSG00000126264	19	hematopoietic cell signal transducer	1	1	2	5	2	618	HCST	0.0	0.0	0.0	0.0	280.9

ENSG00000004468	4	CD38 molecule	1	1	1	1	1	279	CD38	0.0	0.0	0.0	0.0	279.0
ENSG00000147443	8	docking protein 2	1	1	1	1	1	266	DOK2	0.0	0.0	0.0	0.0	266.0
ENSG00000235576	2	0	1	1	3	1	1	362	LINC01871	0.0	0.0	0.0	0.0	258.6
ENSG00000082074	5	FYN binding protein 1	1	1	1	1	1	258	FYB1	0.0	0.0	0.0	0.0	258.0
ENSG00000179144	7	GTPase, IMAP family member 7	1	1	1	1	1	258	GIMAP7	0.0	0.0	0.0	0.0	258.0
