# Supplementary material for: GMP-compliant iPS cell lines show widespread plasticity in a new set of differentiation workflows for cell replacement and cancer immunotherapy
Source: Stem Cells Transl Med. 2024 Jul 23;13(9):898–911. doi: 10.1093/stcltm/szae047 (PMC11386223; doi:10.1093/stcltm/szae047)
Supplement: szae047_suppl_Supplementary_Table_S3 [file szae047_suppl_supplementary_table_s3.pdf]

Antibodies

Flow cytometry

Name / antigen - conjugated Abs	Supplier	Cat.no.	Dilution	Control / remarks
Cardiac troponin C (cTnC)	Miltenyi Biotec	130-119-575	1:50	IgG1-FITC
2B4	Miltenyi Biotec	130-132-358	1:50	IgG1-PE
CD117	Miltenyi Biotec	130-116-609	1:200	IgG1-PE
CD122	Miltenyi Biotec	130-100-251	1:20	IgG1-FITC
CD14	Miltenyi Biotec	130-110-520	1:50	IgG1-APC
CD144 (CDH5)	BD Biosciences	561714	1:20	IgG1-PE
CD16	Miltenyi Biotec	130-113-392	1:50	IgG1-FITC
CD163	Miltenyi Biotec	130-112-286	1:50	IgG1-PE
CD166	Miltenyi Biotec	130-125-116	1:50	IgG1-FITC
CD28	BioLegend	302908	1:20	IgG1-PE
CD31	BioLegend	303104	1:20	IgG1-FITC
CD31 (PECAM-1)	BioLegend	303104	1:20	IgG1-FITC
CD34	BD Pharming	555821	1:20	IgG1-FITC
CD34	Miltenyi Biotec	130-120-514	1:50	IgG1-APC
CD34	BD Pharmingen	555821	1:20	IgG1-FITC
CD38	Miltenyi Biotec	130-126-887	1:50	IgG1-VioBright B515
CD3ε	BioLegend	317318	1:10	IgG1-APC
CD4	BioLegend	317408	1:40	IgG1-FITC
CD43	Miltenyi Biotec	130-114-598	1:200	IgG1-VioBlue
CD45	Miltenyi Biotec	130-110-632	1:50	IgG1-PE
CD47	Miltenyi Biotec	130-123-691	1:20	IgG1-FITC
CD5	Miltenyi Biotec	130-110-990	1:200	IgG1-PE
CD7	Miltenyi Biotec	130-124-932	1:200	IgG1-APC
CD86	Miltenyi Biotec	130-116-264	1:50	IgG1-APC
CD8a	BioLegend	300928	1:20	PacificBlue-conjugated
CD90	Miltenyi Biotec	130-120-514	1:50	IgG1-PE
HLA A/B/C (class I)	Miltenyi Biotec	130-120-432	1:50	IgG1-FITC
HLA DR/DQ/DP (class II)	Miltenyi Biotec	130-123-695	1:50	IgG1-FITC
KDR (FLK1)	Miltenyi Biotec	130-120-620	1:50	IgG1-PE
NKG2D	Miltenyi Biotec	130-111-846	1:50	IgG1-APC
NKp30	Miltenyi Biotec	130-112-431	1:50	IgG1-APC
NKp44	Miltenyi Biotec	130-120-361	1:50	IgG1-VioBright B515
NKp46	Miltenyi Biotec	130-112-121	1:50	IgG1-PE
TCR αβ	Miltenyi Biotec	130-113-536	1:200	APC-Vio770-conjugated
TCR γδ	BioLegend	331217	1:40	BrilliantViolet 421-conjugated
TRA-1-60	Miltenyi Biotec	130-122-965	1:50	IgG1-PE
IgG1-FITC	Miltenyi Biotec	130-113-437	1:50	Gating control for conjugated Abs
IgG1-PE	Miltenyi Biotec	130-113-438	1:50	Gating control for conjugated Abs
IgG1-APC	Miltenyi Biotec	130-113-446	1:50	Gating control for conjugated Abs

IgG1-VioBright B515	Miltenyi Biotec	130-113-457	1:50	Gating control for conjugated Abs
IgG1-VioBlue	Miltenyi Biotec	130-119-881	1:200	Gating control for conjugated Abs
Goat α-mouse IgG 488	Thermo	A11001	1:1000	Secondary Ab & gating control for unconjugated Abs
Rabbit α-mouse IgG 647	Thermo	A21239	1:1000	Secondary Ab & gating control for unconjugated Abs
Goat α-rabbit IgG 647	Thermo	A21244	1:1000	Secondary Ab & gating control for unconjugated Abs

**Name / antigen - unconjugated Abs**	**Supplier**	**Cat.no.**	**Dilution**	**Secondary Ab & gating control / remarks**
BEST1	Abcam	ab2182	1:150	Rabbit α-mouse IgG 647
CD105	BioLegend	323202	1:25	Rabbit α-mouse IgG 647
CD14	Thermo	MA119223	1:30	Goat α-mouse IgG 488
CD44	BD Biosciences	555476	1:30	Goat α-mouse IgG 488
CD73	BD Biosciences	750060	1:20	Goat α-mouse IgG 488
CD90	BioLegend	328102	1:100	Goat α-mouse IgG 488
MITF	Abcam	ab12039	1:150	Rabbit α-mouse IgG 647
PMEL17	Abcam	ab137078	1:50	Goat α-rabbit IgG 647
ZO1	Abcam	ab216880	1:150	Goat α-rabbit IgG 647

Immunocytochemistry

**Primary Abs**	**Supplier**	**Cat.no.**	**Dilution**	**Secondary Ab / remarks**
KDR (FLK1)	Cell Signaling	2479S	1:100	Donkey α-rabbit IgG 488
AFP	Sigma	A8452	1:500	Goat α-mouse IgG 488
BEST1	Abcam	ab2182	1:150	Goat α-mouse IgG 488
Cardiac troponin I (cTnI)	Santa Cruz	sc-15368	1:400	Goat α-rabbit IgG 568
CD31 (PECAM-1)	Abcam	ab9498	1:100	Goat α-mouse IgG 488
CD34	Abcam	ab81289	1:100	Donkey α-rabbit IgG 488
CRALBP	Abcam	ab15051	1:150	Goat α-mouse IgG 488
MITF	Abcam	ab122982	1:150	Goat α-rabbit IgG 568
OCT4	Santa Cruz	sc-5279	1:150	Rabbit α-mouse IgG 568
SMA	Agilent	M085129-2	1:200	Goat α-mouse IgG 488
SOX2	R&D Systems	AF2018	1:200	Rabbit α-goat IgG 568
ZO1	Abcam	ab216880	1:150	Goat α-rabbit IgG 568
α-actinin (ACTN2)	Sigma	A7811	1:800	Goat α-mouse IgG 488
β-III-tubulin (TUBB3)	Sigma	T8660	1:1000	Goat α-mouse IgG 488

**Secondary Abs**	**Supplier**	**Cat.no.**	**Dilution**	**Remarks**
Donkey α-rabbit IgG 488	Thermo	A21206	1:1000	
Goat α-mouse IgG 488	Thermo	A11001	1:1000	
Goat α-rabbit IgG 568	Thermo	A11011	1:1000	
Rabbit α-goat IgG 568	Thermo	A11078	1:1000	
Rabbit α-mouse IgG 568	Thermo	A11061	1:1000	
