# Supplementary material for: GMP-compliant iPS cell lines show widespread plasticity in a new set of differentiation workflows for cell replacement and cancer immunotherapy
Source: Stem Cells Transl Med. 2024 Jul 23;13(9):898–911. doi: 10.1093/stcltm/szae047 (PMC11386223; doi:10.1093/stcltm/szae047)
Supplement: szae047_suppl_Supplementary_Table_S4 [file szae047_suppl_supplementary_table_s4.pdf]

DNA

SYBR Green qPCR primers

Name / amplicon	Fwd primer sequence	Rev primer sequence	Produkt size [bp]	Remarks
RPL37A	GTGGTTCCTGCATGAAGACAGTG	TTCTGATGGCGGACTTTACCG	84	Housekeeping gene for normalization
ACTA2 (SMA)	TGTCCACCGCAAATGCTTCT	AGCTTTGGCTAGGAATGATTTGGA	119	
ACTN2	GCCAGAGAGAAGGATGCAATCAC	AAGCATGGGAACCTGGAATCAA	75	
AFP	AGCAGCTTGGTGGTGGATGA	CCTGAGCTTGGCACAGATCCT	88	
ALB	ACTTGTTGCTGCAAGTCAAGCTG	CTTATTCTCATGGTAGGCTGAGATGCT	77	
ATP1B1	CTGCCCATCACTTTGGCTAGTG	CAGCATGTGATGCCTCCAGAA	95	
BEST1	CCACTGTATCAGAGGCCAGGCTA	ACGGCGCTGATGGTTCTAGG	91	
CD34	TCACCAGCTGCCTCTTCTGTG	AACCAGGATCCCTGCTCAACC	84	
CD47 TG 1	TAGGAAAGCTGTAGAGGAACCC	ATCAGTCAGTCAGTGCAGGAG	98	Transgene-specific CD47, amplicon 1
CD47 TG 2	CTAGGAAAGCTGTAGAGGAACCC	GTCAGTGCAGGAGGAGACAAC	91	Transgene-specific CD47, amplicon 2
CLDN19	CACAATGCCTAGGCTCCAACC	TGACTTCTGATGCCAGGTCCA	70	
DCX	AGGGCTTTCTTGGGTCAGAGG	GCTGCGAATCTTCAGCACTCA	94	
DNMT3B	GCTCACAGGGCCCGATACTT	GCAGTCCTGCAGCTCGAGTTTA	93	
FGF2	GGCAAGATGCAGGAGAGAGGA	GCCACGTGAGAGCAGAGCAT	119	
gDNA control assay	CCCGCTCTCAGGGAGATTTG	CCCTGCCATTGGTTGGAGA	77	Template control for vector elimination assays
miR-302/367 HT	TGGAGGAGAACACGAATCTTTGG	GAACAGGGAAGAGGAAGAGAAGCA	83	
MITF	GACTGTCTCTTGCGGATGCTTG	AGAAATTATTGCTGCATCCAACAGC	74	
MYH6	ACCTGGTGGACAAGCTGCAA	CACCTTGCGGAACTTGGACA	101	
MYH7	TTGATCTGCTCAGCCCTGGA	GCTTCCTCCCAAGGAGCTGTT	74	
MYL2	TGGTCCCTGCCCTCATCTCT	GGCAGCCACATGGCTAACAG	73	
MYL4	CTGGGCAAGAGGATGCCAAT	GCACCTGGAAGACTCTGCTTCA	87	
MYL7 (MLC2a)	CAGATGTTCGCCCTGACACC	TCGTCTCCATGGGTGATGATG	83	
NANOG	AGGTCTCGTATTTGCTGCATCGT	GAAACACTCGGTGAAATCAGGGTAA	119	
NEUROD1	GACTGATTGCACCAGCCCTTC	CGGACGGTTCGTGTTTGAAAG	89	
NKX2.5	ACCGATCCCACCTCAACAGC	CTCCGCAGGAGTGAATGCAA	96	
NPNT	TGGTAAAGGCAGGGCTGGAG	ACAGCCATATATTCTGCCGTTACTCA	70	
OCT4 (POU5F1)	GGAAGGAATTGGGAACACAAAGG	AACTTCACCTTCCCTCCAACCA	71	
OTX2	GCCAATCCTTGGTTGAATCTTAGG	CAATCAGTCACACAATTCACACAGC	120	
PMEL17	AGGTGTTTCTGTCAACTCCAGGAAA	TTGTCCCATTGGTGAGAACAGC	97	
RLPB1	CATCTCCTGCCAGCTGAACTGT	AGCAGCCCTTTCCTAGCCTTG	82	
RPE65	AGCCCGGGCAACTTCACTTA	GTGGTGGTGAGCCCAGGAG	84	
SOX2	TGGCGAACCATCTCTGTGGT	CCAACGGTGTCAACCTGCAT	111	
TNNT2 (CTNT)	GGCAGCTCCTGTTTGGAAATG	TTATTACTGGTGTGGAGTGGGTGTG	87	
TYR	GAACACACCTGTCTTTGTCTTGCTG	CCTCATTACCAAATAGCATCCTTTCC	98	

TaqMan qPCR primers

Name / amplicon	Fwd primer sequence	Rev primer sequence	Produkt size	Remarks
Vector assay 1	GAAGGACATATGGGAGGGCAAA	TTGTTCATGGCAGCCAGCAT	97	Episomal vector elimination assay 1. Probe: TCAGAATGAGTATTTGGTTTAGAGTTTGGCAAC
Vector assay 2	AGCGGATCTCAATTCCGATCATA	CTGGACGTAAACTCCTCTTCAGACCT	100	Episomal vector elimination assay 2. Probe: CAATAACCCTTAATATAACTTCGTATAATGTATGCT

Conventional PCR primers

Name / amplicon	Fwd primer sequence	Rev primer sequence	Produkt size	Remarks
B2M span	GCAGCGCAATCTCCAGTGAC	CCTGCTCAACTGCAGGGAAAC	797 (wt) / 207 (mut)	Spanning CRISPR-induced deletion in gDNA
RFXANK span	TGTGTGTGTGACCGTGTTCATGT	CTCCAGCAGGAAGCGAACG	251 (wt) / 153 (mut)	Spanning CRISPR-induced deletion in gDNA
AAVS1 span	CTCCCTCCCAGGATCCTCTC	CCCCGTTCTCCTGTGGATTC	507 (wt) / 3454 (KI)	Spanning cut site in AAVS1 locus (albeit not spanning KI homology arms)
AAVS/CD47 upstream	TGAGTTTGCCAAGCAGTCAC	GCGTTACTATGGGAACATACGTC	969	Upstream detection of CD47 cassette integration in AAVS1 (spanning homology arm)
AAVS/CD47 upstream	GACACTGTCGTCATTCCATGCTTTG	ACCCATGCAGTCCTCCTTACC	2436	Downstream detection of CD47 cassette integration in AAVS1 (spanning homology arm)

Transgenes

GFP knock-in sequence with homology arms of AAVS1 locus, CAG promoter, CD47 reading frame, and 3' UTR

CTCTCCTGGGCTTGCCAAGGACTCAAACCCAGAAGCCCAGAGCAGGGCCTTAGGGAAGCGGGACCCTGCTCTGGGCGGAGGAATATGTCCCAGATAGCACTGGGGACTCTTTAAGGAAAGAAGGATGGAGAAAGAGAAAGGGAGTAGAGGCGGCCACGACCTGG
TGAACACCTAGGACGCACCATTCTCACAAAGGGAGTTTTCCACACGGACACCCCCCTCCTCACCACAGCCCTGCCAGGACGGGGCTGGCTACTGGCCTTATCTCACAGGTAAAACTGACGCACGGAGGAACAATATAAATTGGGGACTAGAAAGGTGAAGAGCC
AAAGTTAGAACTCAGGACCAACTTATTCTGATTTTGTTTTTCCAAACTGCTTCTCCTCTTGGGAAGTGTAAGGAAGCTGCAGCACCAGGATCAGTGAAACGCACCAGACAGCCGCGTCAGAGCAGCTCAGGTTCTGGGAGAGGGTAGCGCAGGGTGGCCACTGA
GAACCGGGCAGGTCACGCATCCCCCCCTTCCCTCCCACCCCCTGCCAAGCTCTCCCTCCCAGGATCCTCTCTGGCTCCATCGTAAGCAAACCTTAGAGGTTCTGGCAACGTTACATAACTTACGGTAAATGGCCCGCCTGGCTGACCGCCCAACGACCCCCGCC
CATTGACGTCAATAATGACGTATGTTCCCATAGTAACGCCAATAGGGACTTTCCATTGACGTCAATGGGTGGAGTATTTACGGTAAACTGCCCACTTGGCAGTACATCAAGTGTATCATATGCCAAGTACGCCCCCTATTGACGTCAATGACGGTAAATGGCCC
GCCTGGCATTATGCCCAGTACATGACCTTATGGGACTTTCCTACTTGGCAGTACATCTACGTATTAGTCATCGCTATTACCATGGTCGAGGTGAGCCCCACGTTCTGCTTCACTCTCCCCATCTCCCCCCCCTCCCCACCCCCAATTTTGTATTTATTTATTTT
TTAATTATTTTGTGCAGCGATGGGGGCGGGGGGGGGGGGGGGGCGCGCGCCAGGCGGGGCGGGGCGGGGCGAGGGGCGGGGCGGGGCGAGGCGGAGAGGTGCGGCGGCAGCCAATCAGAGCGGCGCGCTCCGAAAGTTTCCTTTTATGGCGAGGCGGCGGCGGC
GGCGGCCCTATAAAAAGCGAAGCGCGCGGCGGGCGGGGAGTCGCTGCGACGCTGCCTTCGCCCCGTGCCCCGCTCCGCCGCCGCCTCGCGCCGCCCGCCCCGGCTCTGACTGACCGCGTTACTCCCACAGGTGAGCGGGCGGGACGGCCCTTCTCCTCCGGGCT
GTAATTAGCGCTTGGTTTAATGACGGCTTGTTTCTTTTCTGTGGCTGCGTGAAAGCCTTGAGGGGCTCCGGGAGGGCCCTTTGTGCGGGGGGAGCGGCTCGGGGGGTGCGTGCGTGTGTGTGTGCGTGGGGAGCGCCGCGTGCGGCTCCGCGCTGCCCGGCGGC
TGTGAGCGCTGCGGGCGCGGCGCGGGGCTTTGTGCGCTCCGCAGTGTGCGCGAGGGGAGCGCGGCCGGGGGCGGTGCCCCGCGGTGCGGGGGGGGCTGCGAGGGGAACAAAGGCTGCGTGCGGGGTGTGTGCGTGGGGGGGTGAGCAGGGGGTGTGGGCGCGTC
GGTCGGGCTGCAACCCCCCCTGCACCCCCCTCCCCGAGTTGCTGAGCACGGCCCGGCTTCGGGTGCGGGGCTCCGTACGGGGCGTGGCGCGGGGCTCGCCGTGCCGGGCGGGGGGTGGCGGCAGGTGGGGGTGCCGGGCGGGGCGGGGCCGCCTCGGGCCGGGG
AGGGCTCGGGGGAGGGGCGCGGCGGCCCCCGGAGCGCCGGCGGCTGTCGAGGCGCGGCGAGCCGCAGCCATTGCCTTTTATGGTAATCGTGCGAGAGGGCGCAGGGACTTCCTTTGTCCCAAATCTGTGCGGAGCCGAAATCTGGGAGGCGCCGCCGCACCCCC
TCTAGCGGGCGCGGGGCGAAGCGGTGCGGCGCCGGCAGGAAGGAAATGGGCGGGGAGGGCCTTCGTGCGTCGCCGCGCCGCCGTCCCCTTCTCCCTCTCCAGCCTCGGGGCTGTCCGCGGGGGGACGGCTGCCTTCGGGGGGGACGGGGCAGGGCGGGGTTCGG
CTTCTGGCGTGTGACCGGCGGCTCTAGAGCCTCTGCTAACCATGTTCATGCCTTCTTCTTTTTCCTACAGCTCCTGGGCAACGTGCTGGTTATTGTGCTGTCTCATCATTTTGGCAAAGAATTGATTTGATACCGCGGGCCCTTCGCCACCATGGTCAGCAAGG
GCGAGGAACTGTTCACCGGGGTGGTGCCCATCCTGGTCGAGCTGGACGGCGACGTAAACGGCCACAAGTTCAGCGTGTCCGGCGAGGGCGAGGGCGATGCCACCTACGGCAAGCTGACCCTGAAGTTCATCTGTACCACCGGCAAGCTGCCCGTGCCCTGGCCC
ACCCTCGTGACCACCCTGACCTACGGCGTGCAATGCTTCAGCCGCTACCCCGACCACATGAAGCAGCACGACTTCTTCAAGTCCGCCATGCCCGAAGGCTACGTCCAGGAGCGCACCATCTTCTTCAAGGACGACGGCAACTACAAGACCCGCGCCGAGGTGAA
GTTCGAGGGCGACACCCTGGTGAACCGCATCGAGCTGAAGGGCATCGACTTCAAGGAGGACGGCAACATCCTGGGGCACAAGCTGGAGTACAACTACAACAGCCACAACGTCTATATCATGGCCGACAAGCAGAAGAACGGCATCAAGGTGAACTTCAAGATCC
GCCACAACATCGAGGACGGCAGCGTGCAACTCGCCGACCACTACCAGCAGAACACCCCCATCGGCGACGGCCCCGTGCTGCTGCCCGACAACCACTACCTGAGCACCCAGTCCGCCCTGAGCAAAGACCCCAACGAGAAGCGCGATCACATGGTCCTGCTGGAG
TTCGTGACCGCCGCCGGGATCACTCTCGGCATGGACGAGCTGTACAAGTAGAAGTTGTCTCCTCCTGCACTGACTGACTGATACAATCGATTTCTGGATCCGCAGGCCTCTGCTAGCTTGACTGACTGAGATACAGCGTACCTTCAGCTCACAGACATGATAAG
ATACATTGATGAGTTTGGACAAACCACAACTAGAATGCAGTGAAAAAAATGCTTTATTTGTGAAATTTGTGATGCTATTGCTTTATTTGTAACCATTATAAGCTGCAATAAACAAGTTAACAACAACAATTGCATTCATTTTATGTTTCAGGTTCAGGGGGAGG
TGTGGGAGGTTTTTTGGAGAGAGATGGCTCCAGGAGGTGGGGGTGTGTCACCAGATAAGGAATCTGCCTAACAGGAGGTGGGGGTTAGACCCAATATCAGGAGACTAGGAAGGAGGAGGCCTAAGGATGGGGCTTTTCTGTCACCAATCCTGTCCCTAGTGGCC
CCACTGTGGGGTGGAGGGGACAGATAAAAGTACCCAGAACCAGAGCCACATTAACCGGCCCTGGGAATATAAGGTGGTCCCAGCTCGGGGACACAGGATCCCTGGAGGCAGCAAACATGCTGTCCTGAAGTGGACATAGGGGCCCGGGTTGGAGGAAGAAGACT
AGCTGAGCTCTCGGACCCCTGGAAGATGCCATGACAGGGGGCTGGAAGAGCTAGCACAGACTAGAGAGGTAAGGGGGGTAGGGGAGCTGCCCAAATGAAAGGAGTGAGAGGTGACCCGAATCCACAGGAGAACGGGGTGTCCAGGCAAAGAAAGCAAGAGGATG
GAGAGGTGGCTAAAGCCAGGGAGACGGGGTACTTTGGGGTTGTCCAGAAAAACGGTGATGATGCAGGCCTACAAGAAGGGGAGGCGGGACGCAAGGGAGACATCCGTCGGAGAAGGCCATCCT

CD47 knock-in sequence with homology arms of AAVS1 locus, CAG promoter, CD47 reading frame, and 3' UTR

CTCTCCTGGGCTTGCCAAGGACTCAAACCCAGAAGCCCAGAGCAGGGCCTTAGGGAAGCGGGACCCTGCTCTGGGCGGAGGAATATGTCCCAGATAGCACTGGGGACTCTTTAAGGAAAGAAGGATGGAGAAAGAGAAAGGGAGTAGAGGCGGCCACGACCTGG
TGAACACCTAGGACGCACCATTCTCACAAAGGGAGTTTTCCACACGGACACCCCCCTCCTCACCACAGCCCTGCCAGGACGGGGCTGGCTACTGGCCTTATCTCACAGGTAAAACTGACGCACGGAGGAACAATATAAATTGGGGACTAGAAAGGTGAAGAGCC
AAAGTTAGAACTCAGGACCAACTTATTCTGATTTTGTTTTTCCAAACTGCTTCTCCTCTTGGGAAGTGTAAGGAAGCTGCAGCACCAGGATCAGTGAAACGCACCAGACAGCCGCGTCAGAGCAGCTCAGGTTCTGGGAGAGGGTAGCGCAGGGTGGCCACTGA
GAACCGGGCAGGTCACGCATCCCCCCCTTCCCTCCCACCCCCTGCCAAGCTCTCCCTCCCAGGATCCTCTCTGGCTCCATCGTAAGCAAACCTTAGAGGTTCTGGCAACGTTACATAACTTACGGTAAATGGCCCGCCTGGCTGACCGCCCAACGACCCCCGCC
CATTGACGTCAATAATGACGTATGTTCCCATAGTAACGCCAATAGGGACTTTCCATTGACGTCAATGGGTGGAGTATTTACGGTAAACTGCCCACTTGGCAGTACATCAAGTGTATCATATGCCAAGTACGCCCCCTATTGACGTCAATGACGGTAAATGGCCC
GCCTGGCATTATGCCCAGTACATGACCTTATGGGACTTTCCTACTTGGCAGTACATCTACGTATTAGTCATCGCTATTACCATGGTCGAGGTGAGCCCCACGTTCTGCTTCACTCTCCCCATCTCCCCCCCCTCCCCACCCCCAATTTTGTATTTATTTATTTT
TTAATTATTTTGTGCAGCGATGGGGGCGGGGGGGGGGGGGGGGCGCGCGCCAGGCGGGGCGGGGCGGGGCGAGGGGCGGGGCGGGGCGAGGCGGAGAGGTGCGGCGGCAGCCAATCAGAGCGGCGCGCTCCGAAAGTTTCCTTTTATGGCGAGGCGGCGGCGGC
GGCGGCCCTATAAAAAGCGAAGCGCGCGGCGGGCGGGGAGTCGCTGCGACGCTGCCTTCGCCCCGTGCCCCGCTCCGCCGCCGCCTCGCGCCGCCCGCCCCGGCTCTGACTGACCGCGTTACTCCCACAGGTGAGCGGGCGGGACGGCCCTTCTCCTCCGGGCT
GTAATTAGCGCTTGGTTTAATGACGGCTTGTTTCTTTTCTGTGGCTGCGTGAAAGCCTTGAGGGGCTCCGGGAGGGCCCTTTGTGCGGGGGGAGCGGCTCGGGGGGTGCGTGCGTGTGTGTGTGCGTGGGGAGCGCCGCGTGCGGCTCCGCGCTGCCCGGCGGC
TGTGAGCGCTGCGGGCGCGGCGCGGGGCTTTGTGCGCTCCGCAGTGTGCGCGAGGGGAGCGCGGCCGGGGGCGGTGCCCCGCGGTGCGGGGGGGGCTGCGAGGGGAACAAAGGCTGCGTGCGGGGTGTGTGCGTGGGGGGGTGAGCAGGGGGTGTGGGCGCGTC
GGTCGGGCTGCAACCCCCCCTGCACCCCCCTCCCCGAGTTGCTGAGCACGGCCCGGCTTCGGGTGCGGGGCTCCGTACGGGGCGTGGCGCGGGGCTCGCCGTGCCGGGCGGGGGGTGGCGGCAGGTGGGGGTGCCGGGCGGGGCGGGGCCGCCTCGGGCCGGGG
AGGGCTCGGGGGAGGGGCGCGGCGGCCCCCGGAGCGCCGGCGGCTGTCGAGGCGCGGCGAGCCGCAGCCATTGCCTTTTATGGTAATCGTGCGAGAGGGCGCAGGGACTTCCTTTGTCCCAAATCTGTGCGGAGCCGAAATCTGGGAGGCGCCGCCGCACCCCC
TCTAGCGGGCGCGGGGCGAAGCGGTGCGGCGCCGGCAGGAAGGAAATGGGCGGGGAGGGCCTTCGTGCGTCGCCGCGCCGCCGTCCCCTTCTCCCTCTCCAGCCTCGGGGCTGTCCGCGGGGGGACGGCTGCCTTCGGGGGGGACGGGGCAGGGCGGGGTTCGG
CTTCTGGCGTGTGACCGGCGGCTCTAGAGCCTCTGCTAACCATGTTCATGCCTTCTTCTTTTTCCTACAGCTCCTGGGCAACGTGCTGGTTATTGTGCTGTCTCATCATTTTGGCAAAGAATTGATTTGATACCGCGGGCCCTTCGCCACCATGTGGCCCCTGG
TAGCGGCGCTGTTGCTGGGCTCGGCGTGCTGCGGATCAGCTCAGCTACTATTTAATAAAACAAAATCTGTAGAATTCACGTTTTGTAATGACACTGTCGTCATTCCATGCTTTGTTACTAATATGGAGGCACAAAACACTACTGAAGTATACGTAAAGTGGAAA
TTTAAAGGAAGAGATATTTACACCTTTGATGGAGCTCTAAACAAGTCCACTGTCCCCACTGACTTTAGTAGTGCAAAAATTGAAGTCTCACAATTACTAAAAGGAGATGCCTCTTTGAAGATGGATAAGAGTGATGCTGTCTCACACACAGGAAACTACACTTG
TGAAGTAACAGAATTAACCAGAGAAGGTGAAACGATCATCGAGCTAAAATATCGTGTTGTTTCATGGTTTTCTCCAAATGAAAATATTCTTATTGTTATTTTCCCAATTTTTGCTATACTCCTGTTCTGGGGACAGTTTGGTATTAAAACACTTAAATATAGAT
CCGGTGGTATGGATGAGAAAACAATTGCTTTACTTGTTGCTGGACTAGTGATCACTGTCATTGTCATTGTTGGAGCCATTCTTTTCGTCCCAGGTGAATATTCATTAAAGAATGCTACTGGCCTTGGTTTAATTGTGACTTCTACAGGGATATTAATATTACTT
CACTACTATGTGTTTAGTACAGCGATTGGATTAACCTCCTTCGTCATTGCCATATTGGTTATTCAGGTGATAGCCTATATCCTCGCTGTGGTTGGACTGAGTCTCTGTATTGCGGCGTGTATACCAATGCATGGCCCTCTTCTGATTTCAGGTTTGAGTATCTT
AGCTCTAGCACAATTACTTGGACTAGTTTATATGAAATTTGTGGCTTCCAATCAGAAGACTATACAACCTCCTAGGAAAGCTGTAGAGGAACCCCTTAATGCATTCAAAGAATCAAAAGGAATGATGAATGATGAATAAAAGTTGTCTCCTCCTGCACTGACTG
ACTGATACAATCGATTTCTGGATCCGCAGGCCTCTGCTAGCTTGACTGACTGAGATACAGCGTACCTTCAGCTCACAGACATGATAAGATACATTGATGAGTTTGGACAAACCACAACTAGAATGCAGTGAAAAAAATGCTTTATTTGTGAAATTTGTGATGCT
ATTGCTTTATTTGTAACCATTATAAGCTGCAATAAACAAGTTAACAACAACAATTGCATTCATTTTATGTTTCAGGTTCAGGGGGAGGTGTGGGAGGTTTTTTGGAGAGAGATGGCTCCAGGAGGTGGGGGTGTGTCACCAGATAAGGAATCTGCCTAACAGGA
GGTGGGGGTTAGACCCAATATCAGGAGACTAGGAAGGAGGAGGCCTAAGGATGGGGCTTTTCTGTCACCAATCCTGTCCCTAGTGGCCCCACTGTGGGGTGGAGGGGACAGATAAAAGTACCCAGAACCAGAGCCACATTAACCGGCCCTGGGAATATAAGGTG
GTCCCAGCTCGGGGACACAGGATCCCTGGAGGCAGCAAACATGCTGTCCTGAAGTGGACATAGGGGCCCGGGTTGGAGGAAGAAGACTAGCTGAGCTCTCGGACCCCTGGAAGATGCCATGACAGGGGGCTGGAAGAGCTAGCACAGACTAGAGAGGTAAGGGG
GGTAGGGGAGCTGCCCAAATGAAAGGAGTGAGAGGTGACCCGAATCCACAGGAGAACGGGGTGTCCAGGCAAAGAAAGCAAGAGGATGGAGAGGTGGCTAAAGCCAGGGAGACGGGGTACTTTGGGGTTGTCCAGAAAAACGGTGATGATGCAGGCCTACAAGA
AGGGGAGGCGGGACGCAAGGGAGACATCCGTCGGAGAAGGCCATCCT

RNA

gRNA sequences

Name	Sequence (excl. PAM)	Sequence for second cut	Remarks
B2M	GAUGACCAAAUGUAAACACU	GAAGUUGACUUACUGAAGAA	
RFXANK	GUCCCCAUUUGGCAGCACUG	GGUCUCAAUCUCUCCAAAGG	
AAVS1	CUGGAGCCAUCUCUCUCCUUG	n/a	
